# Epidemiological trends in enamel hypomineralisation and molar-incisor hypomineralisation: a systematic review and meta-analysis

**DOI:** 10.1007/s00784-025-06411-4

**Published:** 2025-06-02

**Authors:** Nour Ammar, Karl-Ferdinand Fresen, Falk Schwendicke, Jan Kühnisch

**Affiliations:** 1 Department of Conservative Dentistry and Periodontology, University Hospital, Ludwig- Maximilians Universität München, Munich, Germany; 2 Department of Pediatric Dentistry and Dental Public Health, Faculty of Dentistry, Alexandria University, Alexandria, Egypt

**Keywords:** Epidemiology, Dental enamel, Diagnostic techniques, Oral health, Cross-sectional studies, Health surveys

## Abstract

**Objectives:**

To assess the prevalence of enamel hypomineralisation (EH), molar-incisor hypomineralisation (MIH), and hypomineralisation affecting the first permanent molars and incisors (M + IH). It was hypothesized that MIH prevalence had not changed significantly over the last two decades.

**Methods:**

Published literature was screened via PubMed, Embase, Google Scholar, and an extensive hand search. A risk of bias assessment (RoB) was conducted. Prevalence estimates were synthesised in a random-effects meta-analysis and reported at the country-level, continent-level, and globally. Subgroup analyses between different MIH diagnosis indices were conducted. Global MIH prevalence trends between 2000 and 2020 were analysed at 5-year intervals.

**Results:**

Among the 746 retrieved references, 174 were eligible. However, 138 low-to-moderate RoB studies were included. EH global prevalence was estimated at 25.3% (20.0–30.6) across 21 studies from 15 countries. MIH prevalence was 15.5% (14.4–16.6) across 135 studies from 53 countries, with estimates ranging from 0.6 to 46.6%. Among these studies, 60 reported M + IH prevalence, estimated at 6.9% (6.0–7.7). The global MIH prevalence as analysed in 5-year intervals showed a relatively constant prevalence rate with no significant differences across the years. Similarly, MIH prevalence did not differ significantly based on the diagnostic index used.

**Conclusion:**

EH phenotypes remain globally widespread with uneven distributions. Data doesn’t support the postulated change in MIH prevalence. Assessments of all EH phenotypes and data from underrepresented countries are needed for effective dental health monitoring.

**Clinical relevance:**

Oral health professionals should systematically and routinely screen for EH/MIH and discuss it with patients, particularly in high prevalence populations.

**Supplementary Information:**

The online version contains supplementary material available at 10.1007/s00784-025-06411-4.

## Introduction

Enamel hypomineralisation (EH) encompasses various qualitative defects in enamel mineralisation [1]. The demarcated opacities characteristic of EH often present as well-defined asymmetrically distributed lesions with distinct boundaries from the adjacent normal enamel, in contrast to the diffuse, symmetrical appearance of fluorosis lesions which typically follow a chronological distribution pattern. A prevalent form of EH is molar-incisor hypomineralisation (MIH) which manifests either as demarcated opacities, enamel breakdown, atypical restorations, and/or extractions. An MIH diagnosis is established when at least one first permanent molar is affected, with or without the involvement of the incisors [1–3]. In some cases, MIH affects both the permanent molars and incisors simultaneously, leading to an extensive form of the disease that is referred to as M + IH [4]. Beyond the aforementioned ‘index teeth’, EH can principally occur in any other primary or permanent tooth. Consequently, another viable epidemiological measure is the number of individuals with at least one tooth showing EH, regardless of its distribution. As a result, the prevalence of EH is inherently greater than that of MIH or M + IH [4, 5].

One of the earliest systematic reviews on the global prevalence of MIH estimated that it ranges from 2.4 to 40.2% [6]. These findings were later corroborated by a review reporting global prevalences in the same range [7]. Recent meta-analyses have refined these calculations yielding close-ranging prevalence estimates ranging from 12.8% (11.5–14.1) to 14.2% (12.6–15.8) [7–12]. Over the past two decades, the number of cross-sectional studies examining the prevalence of EH, MIH, and M + IH has increased noticeably [13], raising speculation about a potential global rise in MIH prevalence [11, 13, 14]. It remains unclear whether the surge in MIH research has influenced prevalence estimates or whether the global prevalence may have actually changed over time. Additionally, inconsistencies may arise from the use of various diagnostic indices, since several indices are widely available and employed.

Existing reviews often summarize data at the continental or broader regional levels [7–9, 11], frequently overlooking insightful country-specific details. Moreover, the quality of the studies included was frequently not appraised [6–8]. Furthermore, meta-analyses reporting on the prevalence of other phenotypes such as EH or M + IH are lacking in the literature. These knowledge gaps highlight the need for an updated, critical evaluation. Therefore, this systematic review and meta-analysis aimed to: (1) provide a comprehensive update on the global status of EH, MIH, and M + IH with emphasis on study quality assessment, detailed reporting of continent- and country-specific estimates, and various diagnostic indices; and (2) explore the epidemiological trends in global MIH prevalence in cross-sectional studies. The null hypothesis was that there would be no significant difference in the global MIH prevalence over the last two decades.

## Materials and methods

This systematic review was reported according to the PRISMA guidelines [15] and aimed to answer the following research question: “What is the global prevalence of children and young adults affected by EH, MIH, and M + IH?”. This review’s protocol was registered (10.17605/OSF.IO/YEXCQ).

### Literature search

The PubMed, Embase, and Google Scholar databases were searched with no restrictions using relevant keywords for studies published until the end of June 2024. The search queries and results can be found in Appendix I in the supplementary materials. Additionally, an extensive hand search was conducted by reviewing reference lists of included studies, previously published reviews, and journals of interest. Any reviews retrieved through the search were screened for references to additional eligible studies. Articles identified through the hand search were added to the database results All records were exported to the Rayyan platform for the removal of duplicates and screening [16]. Owing to the inconsistent MIH diagnosis criteria in studies published before 2000 and given that they had already been summarized in a systematic review by Jälevik in 2010 [6], these studies were excluded from the review. Thus, this systematic review included studies published from 2000 to June 2024.

### Study screening and eligibility criteria

After removing duplicate references, two independent reviewers screened the titles and abstracts of all identified references. The reviewers evaluated the references for eligibility based on the predefined inclusion and exclusion criteria. Eligible studies were those that reported on the prevalence of EH, MIH, and/or M + IH in the permanent dentition. Only cross-sectional and population-based studies that recruited a representative sample were included. In contrast, studies which recruited participants from a convenience sample, or targeted highly specific nonrepresentative populations were excluded. Studies which exclusively reported on other hypomineralisation phenotypes, including deciduous molar hypomineralisation, hypomineralisation on second primary molars, or rare dental diseases were excluded. Studies that did not mention the index used for diagnosis were not considered. Reviews and conference proceedings were also excluded. If a study’s eligibility could not be determined from the abstract, the full text was obtained. The full texts of the eligible publications were retrieved and assessed according to the inclusion criteria.

Furthermore, to avoid duplicate representation and prevent artificially inflated results, all eligible studies were thoroughly examined for redundant reporting. If multiple publications originated from the same study project, the version with the most comprehensive epidemiological data was included. The reviewers were not blinded to the authors, institutions, or publishing journals of the studies under consideration. Any disagreements were resolved in the work group and all studies were included in consensus.

### Risk of bias assessment

A critical appraisal and risk of bias assessment (RoB) were conducted by two reviewers independently. The RoB assessment followed the recommended Critical Appraisal Tool for prevalence studies from the Joanna Briggs Institute (JBI) [17, 18]. This tool allows comprehensive appraisal of the methodological quality of studies reporting prevalence using nine signalling questions that cover study design, conduct, and data analysis (Appendix II). Each question could be answered as yes, no, unclear, or not applicable. In detail, the questions assessed the sampling frame, participant recruitment, sample size calculations, description of the study participants and setting, coverage of the sample, target condition identification/diagnosis methods, use of the identification methods, data analysis, and response rates. An overall appraisal and a recommendation (include, exclude, or seek further information) were given to each study. Studies that showed high RoB in core appraisal items which included failing to use a reliable/standardized MIH diagnosis method, recruiting participants from a convenience sample, or targeting highly specified nonrepresentative populations were excluded (Appendix III). Recognized MIH indices included the European Academy of Paediatric Dentistry (EAPD) index, Developmental Dental Defects of Enamel (DDE) index, Ghanim et al., and other indices that align with that of the EAPD: including the criteria by Cho et al., Wetzel and Reckel, Molar-Incisor Hypomineralisation Severity Scoring System (MIH-SSS), Mathu-Muju and Wright criteria, Kemoli 2008, Cabral 2017, Alaluusua et al., MIH treatment need index (MIH-TNI), and Koch et al. All included studies underwent the RoB assessment. Any study for which the full text was inaccessible was excluded from further analysis.

### Data extraction

Only studies that met the inclusion criteria and showed low or moderate RoB were considered for further analysis. Two reviewers independently extracted the data from the included studies in a structured extraction form in Excel (Excel 2019, Microsoft, Redmond, WA, USA). If necessary, the study authors were contacted for clarification or missing information. In brief, the following data were extracted: bibliographic information, country, continent, examination year, sample size, sample age, study setting, diagnostic index used for scoring, and prevalence results for EH, MIH, and M + IH. The forms were cross-checked between the reviewers, and any disagreements or inconsistent findings were resolved by discussion with the principal investigator.

### Statistical analysis

A systematic approach to the statistical analysis was adopted. Three independent random effects meta-analyses of prevalence for EH, MIH, and M + IH were conducted, and forest plots were created. Owing to the large number of cross-sectional studies, prevalence data were aggregated in subgroup analyses at the country, continent, and global level, respectively. The global prevalence of the different hypomineralisation phenotypes by country was depicted using a heatmap, and the temporal distribution of studies was presented in a scatterplot. Furthermore, a subgroup analysis comparing the MIH prevalence rates reported in studies using the EAPD index [3], the DDE index [19], or other scoring indices was performed.

To investigate trends in MIH prevalence over time, a three-pronged strategy was developed. The included studies were structured into three categories on the basis of publication year, reported examination year, and estimated examination year. Since the exploratory data analysis revealed that only 65 (47.1%) of the studies reported the actual examination year, an estimation was necessary to avoid excluding the majority of the dataset from this analysis. The examination year was estimated based on an average time lag of 3 years between study conduct and publication, as calculated from studies that reported their examination year. Therefore, for studies lacking these data, the estimated examination year was assumed to be 3 years prior to the publication year. For each of the three categories, a random-effects meta-analysis was conducted with subgroups based on four 5-years intervals. Each interval’s meta-analytic MIH prevalence estimate, along with its 95% confidence interval (95% CI), was reported. The p-values for each meta-analysis were also provided. A line graph featuring the meta-analytic estimates for the time intervals across categories was created to illustrate the trends and fluctuations. Sensitivity analyses were conducted. Analyses and illustrations were done using Stata (StataCorp. 2019, release 16, College Station, TX: StataCorp LLC, USA) along with the statistical package metaprop [20].

## Results

**Fig. 1:**
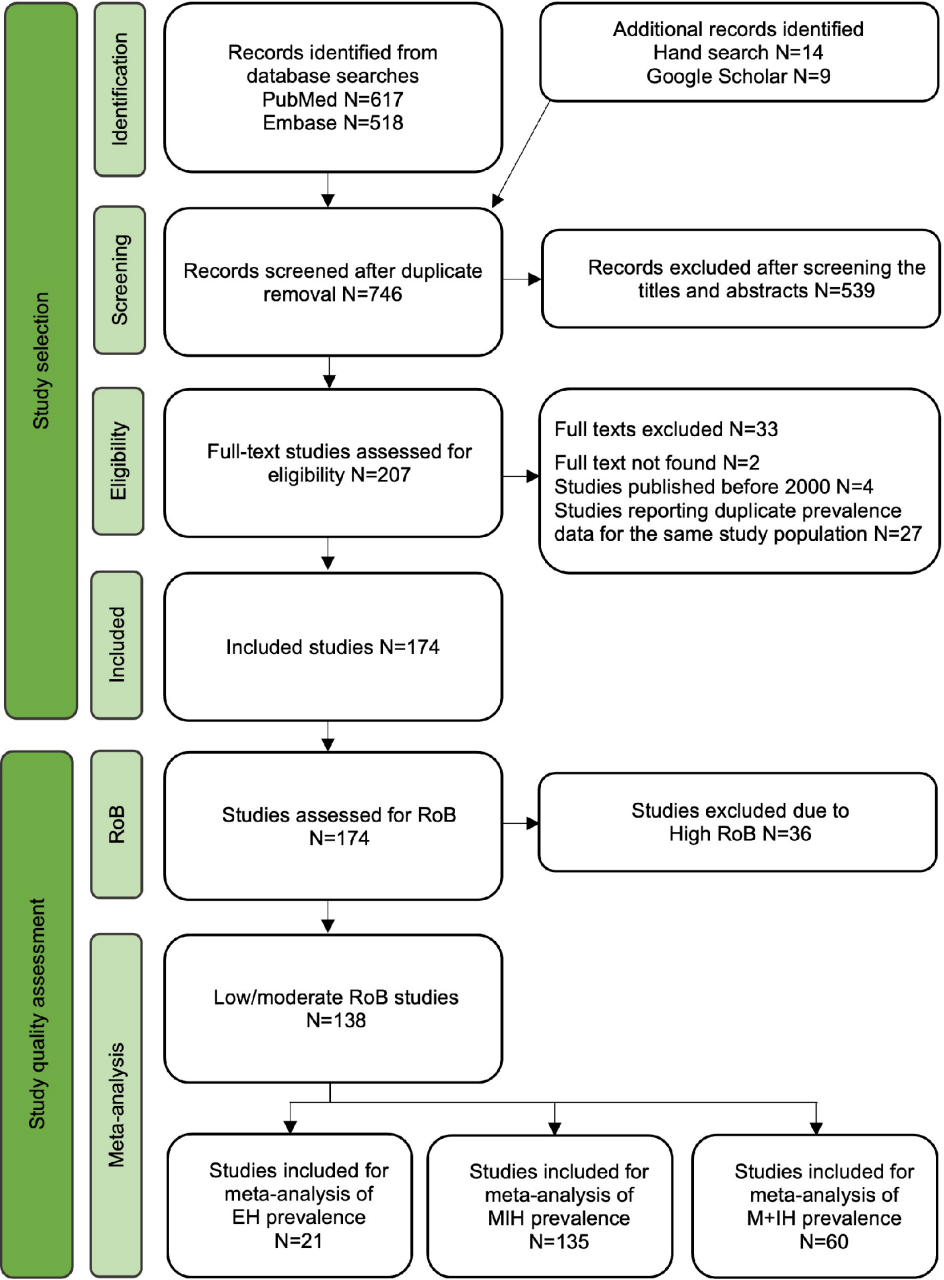
Flowchart of the study selection process.

The literature search yielded 746 records to be screened. After examination of the titles and abstracts 539 did not meet the inclusion criteria. Upon reading the full texts of eligible studies another 33 studies were excluded: 27 articles were found to report on the same study population as another eligible article, four studies were published before 2000 and two articles could not be accessed. At this selection stage 174 cross-sectional epidemiological studies were identified (Fig. 1). In addition, the RoB assessment revealed that 36 (20.7%) studies showed high RoB and were thus excluded. The reasons for study exclusion are summarized in Appendix III. Finally, 138 studies were considered for data extraction and meta-analysis [21–157]. Appendix IV summarizes the data extracted from the included studies.

**Fig. 2:**
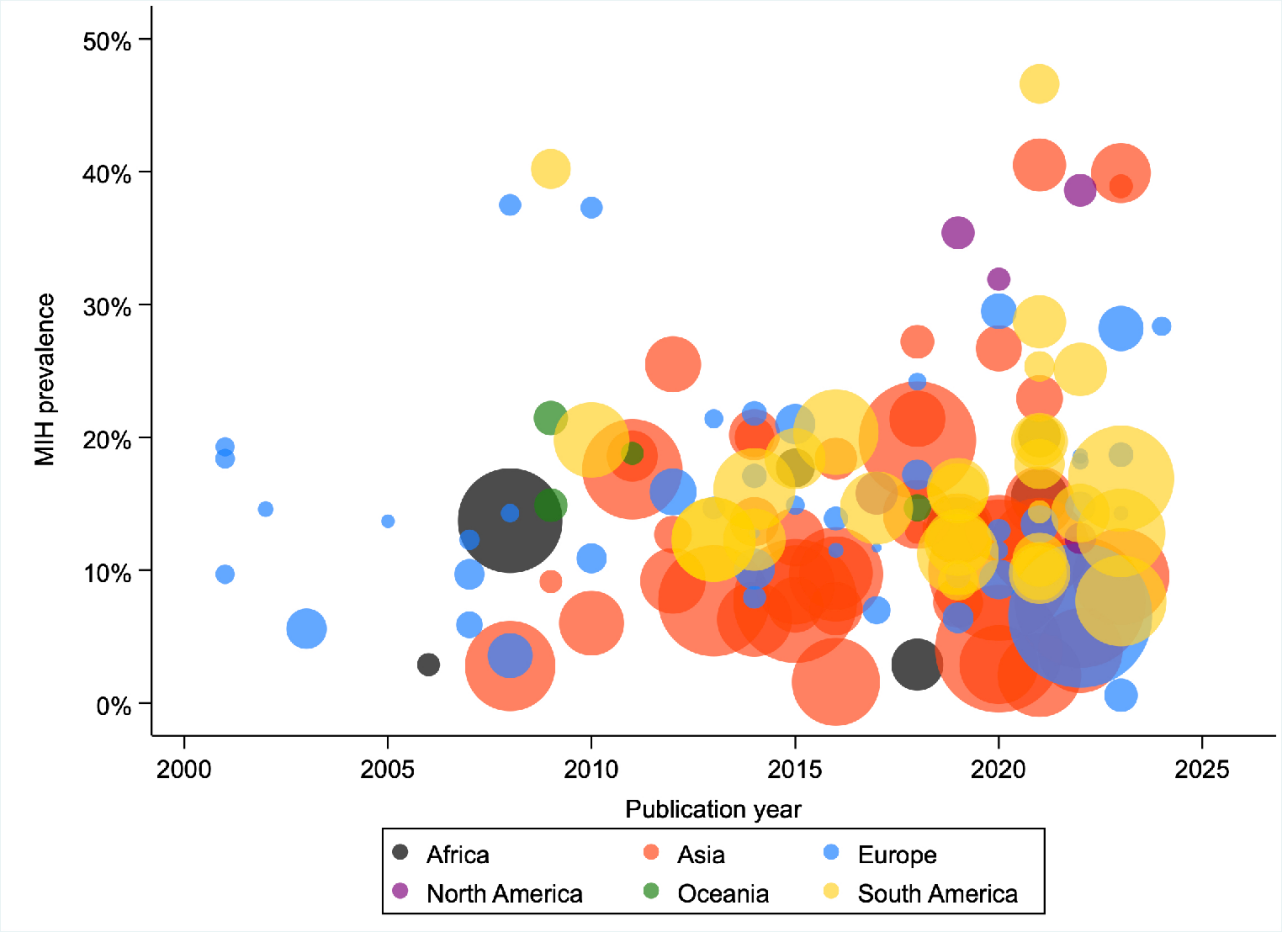
Scatterplot of the temporal spread of publications with reported MIH prevalence as categorized by continent. The size of the circles corresponds to the sample size.

The majority of studies examined children or adolescents with mixed dentition, where participants’ age ranged from 3 to 18 years old. The sample size ranged from 77 to 32,142. MIH prevalence varied widely across the included studies, with estimates as low as 0.6% in Poland [158] and up to 46.6% in Brazil [22] (Fig. 2). Asia (*N* = 44) and Europe (*N* = 45) were the two continents with the highest number of publications (Table 1). All 138 studies were included in the meta-analyses, aggregating the data from a total of 199,662 participants. In summary, 21 studies from 15 countries reported on the prevalence of EH. While 135 studies from 53 countries reported on MIH prevalence, only 60 studies (across 33 countries) reported the M + IH prevalence concomitantly (Fig. 1).

**Table 1:** Meta-analysis results for the prevalence of EH, MIH, and M + IH in included studies per country, continent, and globally. The number of studies reporting on MIH prevalence is indicated for each country and continent next to its name

Continent	Country	EH prevalence	MIH prevalence	M + IH prevalence
Europe (N = 45)	Switzerland (N = 2)	0.075 (0.072–0.078)	0.077 (0.074–0.079)	
	Netherlands (N = 2)		0.114 (0.094–0.135)	0.030 (0.018–0.049)
	Bulgaria (N = 1)		0.036 (0.030–0.043)	
	Norway (N = 2)		0.238 (0.225–0.251)	0.058 (0.044–0.076)
	United Kingdom (N = 2)		0.165 (0.152–0.177)	
	Germany (N = 6)	0.276 (0.021–0.532)	0.111 (0.075–0.147)	0.050 (0.027–0.074)
	Lithuania (N = 1)	0.144 (0.126–0.164)	0.094 (0.080–0.111)	0.021 (0.015–0.031)
	Austria (N = 2)		0.087 (0.076–0.098)	0.035 (0.027–0.042)
	Sweden (N = 1)	0.333 (0.294–0.375)	0.184 (0.153–0.220)	0.087 (0.066–0.115)
	Romania (N = 1)		0.143 (0.106–0.190)	
	Italy (N = 2)	0.269 (0.215–0.330)	0.161 (0.131–0.192)	0.057 (0.038–0.076)
	France (N = 1)		0.187 (0.162–0.214)	
	Spain (N = 4)	0.459 (0.433–0.485)	0.205 (0.105–0.306)	0.141 (0.122–0.160)
	Poland (N = 4)	0.131 (0.117–0.145)	0.077 (0.021–0.133)	0.040 (0.032–0.049)
	Slovenia (N = 1)		0.220 (0.185–0.259)	0.104 (0.080–0.135)
	Bosnia and Herzegovina (N = 4)	0.291 (0.257–0.324)	0.120 (0.102–0.138)	0.086 (0.056–0.116)
	Greece (N = 1)		0.213 (0.197–0.230)	0.114 (0.102–0.128)
	Croatia (N = 1)		0.130 (0.108–0.157)	0.066 (0.050–0.086)
	Belgium (N = 1)	0.217 (0.174–0.268)	0.186 (0.146–0.235)	
	Denmark (N = 3)		0.345 (0.287–0.403)	0.097 (0.077–0.122)
	Finland (N = 3)		0.161 (0.119–0.202)	0.063 (0.040–0.097)
	**Europe estimate**	**0.246 (0.184–0.309)**	**0.154 (0.134–0.174)**	**0.071 (0.057–0.085)**
Asia (N = 44)	United Arab Emirates (N = 3)		0.243 (0.057–0.429)	0.094 (0.067–0.129)
	Iran (N = 3)	0.530 (0.490–0.569)	0.172 (0.127–0.217)	
	Syria (N = 1)		0.397 (0.369–0.426)	
	Saudi Arabia (N = 2)		0.212 (0.196–0.227)	0.121 (0.106–0.138)
	Lebanon (N = 2)		0.264 (0.240–0.288)	0.169 (0.150–0.189)
	India (N = 19)	0.213 (0.201–0.225)	0.081 (0.065–0.098)	0.038 (0.024–0.052)
	Turkey (N = 3)		0.104 (0.054–0.154)	0.059 (0.031–0.108)
	Singapore (N = 1)		0.125 (0.106–0.146)	0.028 (0.020–0.040)
	South Korea (N = 1)		0.060 (0.049–0.074)	
	Thailand (N = 1)		0.196 (0.163–0.234)	0.019 (0.010–0.035)
	Japan (N = 1)		0.198 (0.187–0.210)	
	Iraq (N = 1)		0.186 (0.161–0.214)	0.090 (0.072–0.111)
	Nepal (N = 1)		0.137 (0.114–0.163)	0.116 (0.095–0.141)
	China (N = 3)		0.116 (0.044–0.189)	0.021 (0.018–0.024)
	Jordan (N = 2)		0.160 (0.150–0.171)	0.060 (0.053–0.069)
	**Asia estimate**	**0.326 (0.138–0.514)**	**0.137 (0.119–0.155)**	**0.061 (0.049–0.073)**
South America (N = 29)	Peru (N = 1)		0.198 (0.162–0.240)	
	Brazil (N = 19)		0.178 (0.150–0.207)	0.077 (0.040–0.115)
	Argentina (N = 1^*^)		0.161 (0.140–0.184)	
	Uruguay (N = 1^*^)		0.123 (0.100–0.151)	
	Ecuador (N = 1)		0.092 (0.062–0.135)	
	Chile (N = 2)		0.136 (0.121–0.152)	
	Colombia (N = 3)		0.169 (0.068–0.270)	
	Venezuela (N = 1)		0.254 (0.189–0.331)	
	**South America estimate**		**0.171 (0.151–0.192)**	**0.077 (0.040–0.115)**
North America (N = 6)	United States of America (N = 1)		0.096 (0.070–0.130)	0.051 (0.033–0.078)
	Mexico (N = 5)		0.268 (0.161–0.374)	
	**North America estimate**		**0.239 (0.144–0.334)**	**0.051 (0.033–0.078**)
Africa (N = 7)	Sudan (N = 1)		0.201 (0.170–0.236)	0.125 (0.100–0.155)
	Kenya (N = 1)		0.137 (0.126–0.149)	0.092 (0.083–0.102)
	Nigeria (N = 2)	0.076 (0.060–0.096)	0.044 (0.033–0.054)	0.098 (0.074–0.128)
	Libya (N = 2)	0.091 (0.055–0.147)	0.136 (0.117–0.155)	0.076 (0.062–0.094)
	Egypt (N = 1)		0.131 (0.114–0.150)	
	**Africa estimate**	**0.078 (0.061–0.095)**	**0.128 (0.079–0.177)**	**0.095 (0.079–0.111)**
Oceania (N = 4)	Australia (N = 2)	0.428 (0.397–0.460)	0.184 (0.159–0.210)	
	New Zealand (N = 2)	0.153 (0.125–0.186)	0.160 (0.134–0.186)	0.037 (0.023–0.050)
	**Oceania estimate**	**0.323 (0.081–0.565)**	**0.174 (0.139–0.208)**	**0.037 (0.023–0.050)**
**Global estimate**		**0.253 (0.200–0.306)**	**0.155 (0.144–0.166)**	**0.069 (0.060–0.077)**

*Results for these countries were reported in a single study but were treated as distinct datasets in the meta-analysis

**Fig. 3:**
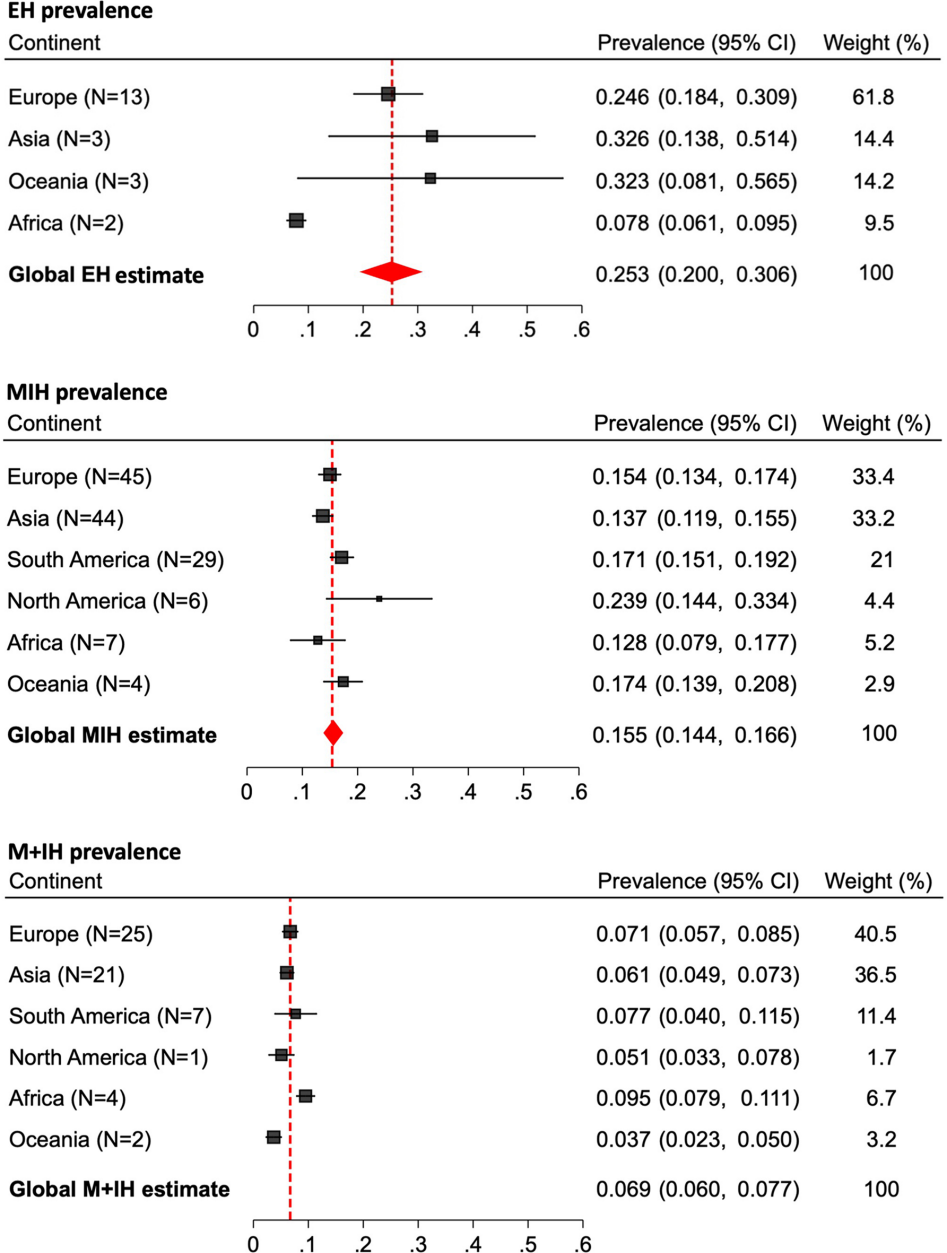
Forest plots for the subgroup analyses of EH, MIH, and M + IH prevalence per continent.

**Fig. 4:**
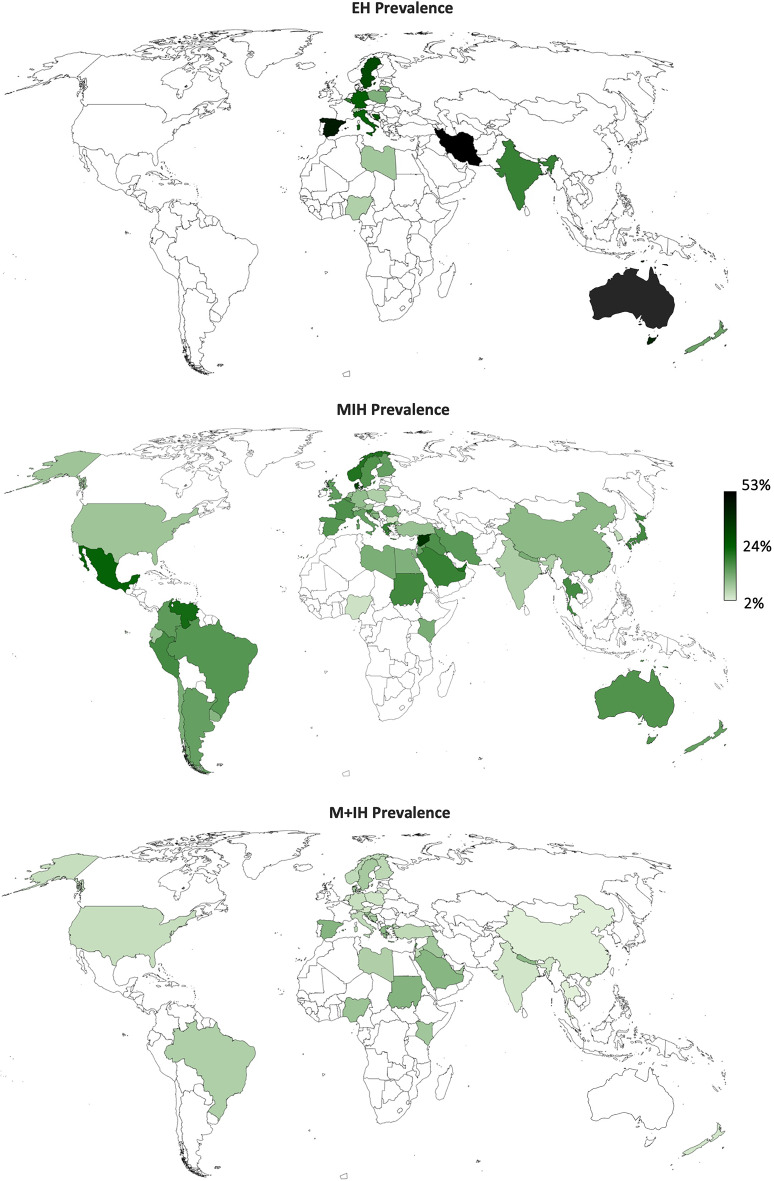
World map showing the estimated prevalence of EH (15 countries), MIH (53 countries), and M + IH (33 countries) per country.

Pooled meta-analysis results for EH, MIH, and M + IH at the country- and continent-levels can be found in Table 1; Fig. 3; the world map (Fig. 4) further facilitates comparison between countries. The pooled global prevalence of EH, MIH, and M + IH were estimated at 25.3% (20.0–30.6), 15.5% (14.4–16.6), and 6.9% (6.0–7.7), respectively. Subgroup analysis by continent (Fig. 3) revealed that Africa had the lowest MIH prevalence at an estimated 12.8% (7.9–17.7), whereas North America had the highest prevalence at 23.9% (14.44–33.4).

The EAPD criteria were the most frequently used MIH diagnostic index (N = 99 studies) followed by the DDE (N = 12). The remaining studies (N = 24) utilized various other indices, with some incorporating multiple indices in their diagnostic process. These included: Ghanim et al. (N = 12), Cho et al. (N = 2), Wetzel and Reckel (N = 2), Molar-Incisor Hypomineralisation Severity Scoring System (MIH-SSS) (N = 2), Mathu-Muju and Wright criteria (N = 1), Kemoli 2008 (N = 1), Cabral 2017 (N = 1), Alaluusua et al. (N = 1), MIH treatment need index (MIH-TNI) (N = 1), and Koch et al. (N = 1). The subgroup comparison between studies using the EAPD, DDE, or other scoring indices revealed no significant differences in the MIH prevalence among studies (15.9% (14.4–17.3), 16.2% (12.7–19.7), and 14.4% (12.1–16.6), respectively; *p* = 0.496).

**Fig. 5:**
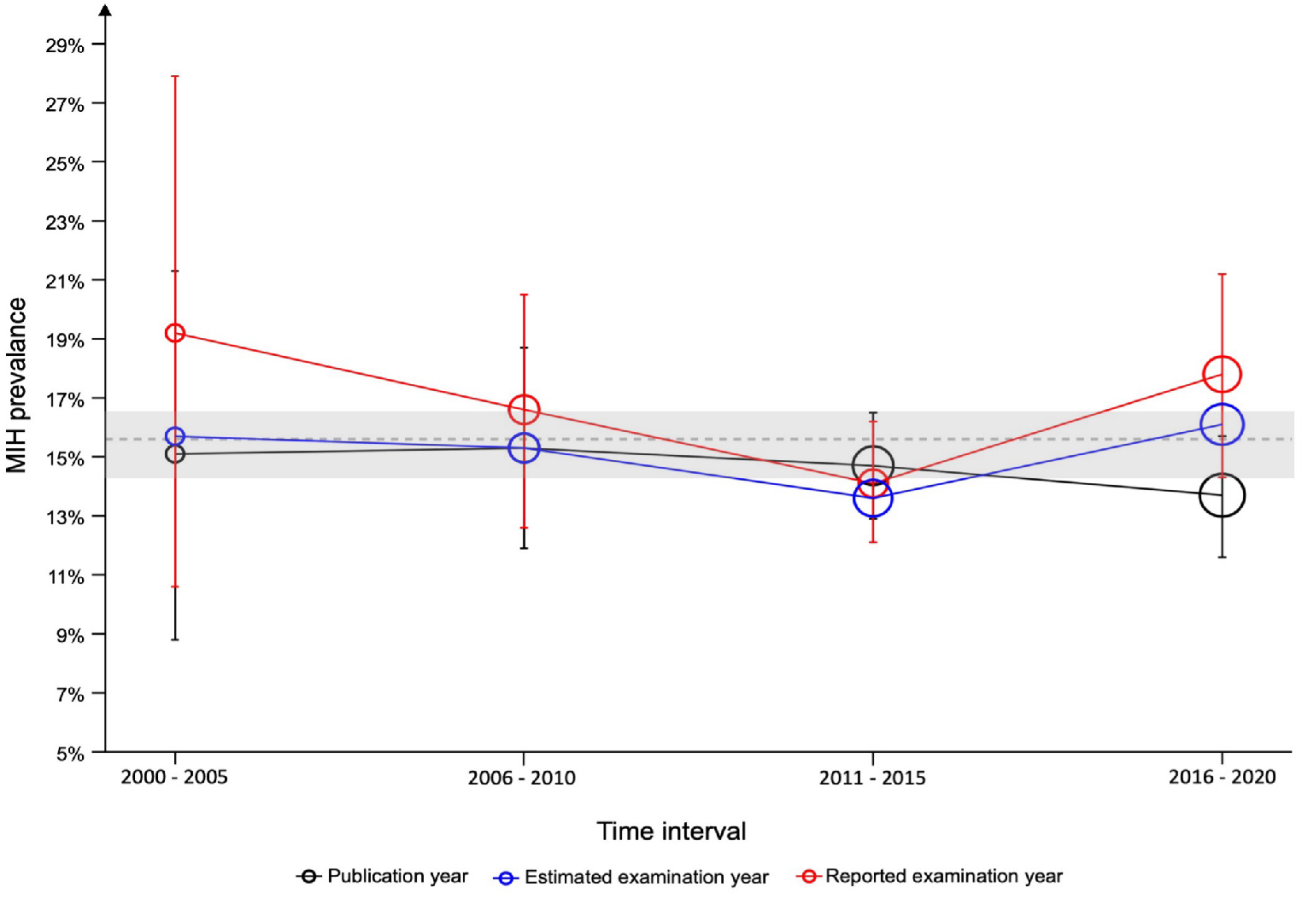
Epidemiological trends of the meta-analytic estimates for MIH prevalence in 5-year intervals as categorized by the publication, estimated examination, and reported examination year. The plotted circles reflect the fluctuations in prevalence estimates and associated 95% confidence intervals for each interval within the three categories. The 95% CI are shown with corresponding coloured error bars. The size of the circles is proportional to the number of studies in this interval. The dashed horizontal reference line and the surrounding grey area represent the estimated MIH global prevalence of 15.5% and the 95% CI (14.4–16.6) as calculated from all included studies.

The examination of the epidemiological trends over the past two decades revealed harmonious trends across the three scenarios (Table 2). When categorized according to year of publication, the prevalence of MIH showed non-significant variation, starting at 15.1% in 2000–2005 and ending at 13.7% in 2016–2020. Similarly, when the reported year of examination was considered, the prevalence initially declined from 19.2% in 2000–2005 to 14.1% in 2011–2015, before increasing to 17.8%. Finally, for studies grouped by the estimated year of examination, the MIH prevalence fluctuated the least, with values ranging from 15.7 to 16.1% (Table 2). These meta-analytic findings showed that while there are minor differences in prevalence over time, there has been no significant shift over the past two decades (*p* ≥ 0.05), as presented in Fig. 5. Sensitivity and subgroup analyses (grouping by country, continent, hypomineralisation phenotype, diagnosis index, and time intervals) were performed and confirmed the robustness of the present findings.

**Table 2:** Meta-analysis results for the prevalence of MIH globally in 5-year intervals. The number of studies used to calculate each estimate is indicated in brackets

Global MIH prevalence according to	2000–2005	2006–2010	2011–2015	2016–2020	*p*-value
\Year of publication (N = 92)	15.1% (8.8–21.3) N = 6	15.3% (11.9–18.7) N = 17	14.7% (12.9–16.5) N = 31	13.7% (11.6–15.7) N = 38	0.809
Reported year of examination (N = 65)	19.2% (10.6–27.9) N = 6	16.6% (12.6–20.5) N = 17	14.1% (12.1–16.2) N = 16	17.8% (14.3–21.2) N = 26	0.221
Estimated year of examination (N = 127)	15.7% (10.0–21.5) N = 9	15.3% (12.6–18.1) N = 26	13.6% (11.7–15.4) N = 41	16.1% (14.3–17.8) N = 51	0.284

MIH prevalence was not calculated for studies before 2000 due to the low number of studies and inconsistent diagnosis criteria. Similarly, calculations were not performed for studies after 2020 because many clinical trials were cancelled or postponed in 20212023 due to the COVID-19 pandemic. Additionally, this interval did not meet the required 5-year observation period making it incomparable to earlier intervals

## Discussion

The expanding body of literature investigating the aetiology, prevalence, and management of EH/MIH in recent decades underscores the growing interest within the dental community [13]. To address the expanding body of literature, this systematic review and meta-analysis critically appraised and synthesized the global evidence on the prevalence of EH, MIH and M + IH, along with detailed reporting of continent- and country-specific data and a comparison of different diagnostic indices. Importantly, the present study explored the epidemiological trends in MIH prevalence over the past two decades, where no significant differences have been found. Thus, the present study’s hypothesis was accepted.

This key finding of the meta-analysis is the notable stability of MIH prevalence over time, contrary to prior speculations of an increasing prevalence [11, 13, 14] (Fig. 5). The random effects sub-group meta-analysis by time intervals exhibited statistically insignificant fluctuations, a trend that was consistent across estimates according to the publication year, estimated examination year, and reported examination year (Table 2). In particular, when it was calculated based on the estimated examination year, the MIH prevalence remained consistently aligned with the global prevalence (Fig. 5). These findings are in line with a recent investigation by Sluka et al. [11], who also reported no evidence of a global increase in prevalence. In contrast to the present study, Sluka et al. approached the data by grouping studies in three birth cohorts based on the ages of the study participants. They reported estimates ranging from 9 to 18% across the cohorts, with no significant differences between the subgroups. However, in one sub-group analysis of 11 studies, they observed a two-fold increase in prevalence between 2000 and 2010. Owing to the complexity of their data, Sluka et al. stated that a meta-analysis was not feasible, and prospective studies are needed to confirm these findings. Despite the different analytical approaches, the epidemiological trends documented in both the present analysis and Sluka et al.’s were consistent [11]. Based on the available cross-sectional MIH data, it can be concluded that there was no increase in the global prevalence thus far. Nevertheless, to truly confirm any changes in MIH or EH prevalence over time, prospectively designed cross-sectional studies that are repeated at standardized time intervals with highly matched cohorts are essential to ascertain such a possibility and enable dental health monitoring.

In addition to investigating epidemiologic trends, this meta-analysis synthesized the global prevalence of three prevalent hypomineralisation phenotypes (Table 2; Fig. 5). The global MIH prevalence was estimated at 15.5% (14.4–16.5). When interpreting the present data in light of the CIs and comparing them to estimates from previous systematic reviews, a consistent trend emerges: the results of the present study and prior estimates closely overlap within the same CI range. In detail, previous meta-analytic estimates include 14.2% (12.6–15.8) [8], 13.5% (12.0–15.1) [10], 13.1% (11.8–14.5) [12], 12.9% (11.7–14.3) [9], and 12.8% (11.5–14.1) [11]. This overlap in CIs reinforces the current findings. It is further supported by the data from Fig. 5, which illustrates that nearly all prevalence estimates lie within or very near the estimated 95% CI (horizontal gray bar). This global estimate is further strengthened by the inclusion of a large number of studies with acceptable RoB compared to previous analyses, as well as the conduction of multiple subgroup analyses, making it a potentially robust estimate.

Consistent with previous reviews reporting MIH prevalence [8, 11, 12], there are significant differences across continents (Fig. 3) alongside considerable variation in prevalence reported across countries (Fig. 4). This review supports previous findings in that North America has the highest MIH prevalence (23.9%) [10, 12], followed by South America (17.1%) (Fig. 3). Similarly, Zhao et al. [8] estimated the prevalence in South America at 18.0%, whereas Lopes et al. [10] reported a combined prevalence for the Americas based on 30 studies at 15.3%. For Africa, this review documented the lowest MIH prevalence at 12.8%, which is comparable to previous estimates of 10.9% and 14.5% [8, 10]. As consistently observed in earlier reviews Europe had the highest number of studies on MIH prevalence, with the present estimate at 15.4%, closely aligning with previous estimates of 14.3% and 14.4% [8, 10]. However, the unequal number of studies across continents calls for cautious interpretation of summary statistics, especially in regions with limited data and high prevalence estimates such as North America and Oceania (Table 1).

While previous reviews have focused predominantly on continent- or superregional-level data, this review uniquely presents country-level meta-analyses for the three hypomineralisation phenotypes (Table 1), which had not been previously calculated in this manner. Notably, Zhao et al. [8] had previously reported the meta-analytic MIH estimates for six countries only. These findings are highly consistent with the present findings, with both reviews’ estimates falling within ± 1%. With data from 53 countries, the present review offers a valuable reference point for assessing prevalence changes at the country level. In contrast, the most comprehensive prior reviews included data from 43 to 49 countries [8, 12].

Part of the novelty of this systematic review and meta-analysis lies in synthesizing the global prevalence of EH and M + IH in a meta-analysis and reporting these findings at the continent- and country-levels (Table 1; Fig. 3). The EH prevalence was estimated at 25.3% (20.0–30.6), with the greatest prevalence reported in Oceania (32.3%). The present search did not yield any studies on the prevalence of EH in the North or South American continents, although these continents showed the highest MIH prevalence globally. Consistent with their high MIH prevalence, the highest EH prevalence was reported in Spain (45.9%) and Iran (53.0%). The global M + IH prevalence was estimated at 6.9% (6.0–7.7). Despite having the lowest MIH prevalence, Africa had the highest prevalence of M + IH at 9.5%. Similar to the data on EH, we found only one study reporting on the prevalence of M + IH in each of North America, South America, and Oceania, which limits the generalizability of the estimates for these continents. Consequently, owing to the lack of comparable studies synthesizing the global estimates of EH or M + IH, comparison with other studies was not possible. However, data from a previous review allow for an inferred estimate of M + IH prevalence at 5.7%, which was calculated based on 36 studies [10], in contrast to the 60 studies accounted for in the present review’s estimate. By synthesizing this data, the review not only bridges a significant gap in the literature but also establishes a reference value for the EH and M + IH phenotypes.

This review analysed 138 cross-sectional studies published after 2000. As has been the case in previous MIH reviews [10–12], studies conducted before 2000 were excluded mainly because of inconsistencies in diagnostic criteria prior to the formal adoption of the EAPD index guidelines in 2003 [3]. The qualitative assessment revealed that 20.7% of the eligible studies had high RoB, which is highly similar to the results of Sluka et al. [11] who reported that 18.6% of the studies were of low quality. An examination into the body of data, as illustrated by the scatterplot in Fig. 2, reveals several key insights. Firstly, it clearly illustrates the increase in the number and size of MIH epidemiological studies over time as confirmed by a recent bibliometric analysis [13]. Moreover, studies with a larger sample size tended to report lower MIH estimates. Furthermore, studies from Asia tend to have larger sample sizes on average. Although Europe was the continent with the most epidemiological studies, the sample sizes recruited in Asian studies were approximately 1.8 times larger than those in European studies (with the exclusion of one health records-based study from Europe [37]). The scatterplot also illustrates the scarcity of data from Africa, North America, and Oceania which highlights future research needs.

The strengths of this systematic review and meta-analysis include the well-defined database search, rigorous study selection, and the critical appraisal process that aimed to minimize potential sources of bias (Appendices II and III). Furthermore, it bridges a gap in the literature by reporting the continent- and country-specific meta-analytic evaluation (Table 1; Fig. 3) as well as investigating the postulated MIH prevalence changes with an evidence-based approach (Table 2; Fig. 5). Despite the extensive efforts to include all eligible studies by thorough hand search, studies published in other languages or indexed in less commonly searched databases may have been overlooked. Methodological variations within the studies included in the review presented challenges for qualitative and quantitative data synthesis. In particular, the complexity and variability of the investigated hypomineralisation phenotypes may have complicated the interpretation of the findings. Only 44.4% of the included studies investigating MIH prevalence reported on M + IH status, often using inconsistent terminology or failing to explicitly report it, which required reviewers to deduct or calculate the needed data from tables, if possible. This highlights an issue with incomplete reporting, potentially causing trial investigators to overlook valuable data that could have been collected and reported at minimal additional effort. Clinically, discerning between the various phenotypes is challenging even for experienced epidemiologists, as it requires thorough documentation EH occurrences across the entire dentition. This comprehensive documentation is vital for accurate statistical exploration. However, for simplicity, some surveys assess only the index teeth, which limits the scope for further analysis. In principle, heterogeneity with regard to EH, MIH, and M + IH prevalence worldwide is expected due to the inherent limits of individual reports and are likely to have a potential influence on the results obtained. This can be attributed to various sources of bias, including differences in the diagnostic acuity of the investigators, inter- and intra-examiner reliability, regional deviations (urban or rural communities), and that the data– even from that from the same country– may represent diverse ethnic populations affecting generalizability of the results to a country, and investigations spanning extended time periods.

Another limitation to consider is study participants’ age. The EAPD recommends the MIH examination to occur at or after 8 years of age, to ensure the complete eruption of the index teeth [1, 3]. However, a definitive diagnosis of EH in the permanent dentition is ideally made after the age of 12, when the eruption status of the teeth is fully established. While the majority of studies used the EAPD criteria, several of which recruited children as young as 4- and 6-years-old, a period during which the index teeth may not have fully erupted, potentially causing an underestimation of the condition. Additionally, many studies assessed cohorts within a broad age range, sometimes extending up to 10 years age difference between participants [23, 117, 159], which may have influenced estimates and contributed to variability in the findings. Therefore, future cross-sectional studies should be designed in compliance with these recommendations [3].

While the EAPD criteria were used in 73.3% of the included studies, this was not always the case. For the recording of EH or MIH, several methods or indices have been recommended in recent decades. In the 2009 review by Jälevik [6], an equal number of studies used the EAPD and mDDE criteria. However, a 2018 review found that the majority of published studies employed the EAPD criteria, a trend which continues to date [12]. The use of varying diagnostic indices can complicate study comparability and potentially lead to differing prevalence estimates. Some reviews concluded that studies employing the EAPD index often report a significantly higher MIH prevalence as compared to those using other indices [10, 12]. However, the present subgroup meta-analysis found no significant differences in global MIH prevalence across different indices (*p* = 0.628), suggesting that overall estimates remain consistent despite variations in diagnostic criteria, and is in agreement with Sluka et al. [11] in this regard.

The present systematic review found no significant change in the global prevalence of MIH. It is plausible that the growing clinical recognition and awareness of MIH in the decades following its formal recognition by the EAPD could have contributed to perceptions of rising prevalence, without necessarily reflecting a true change in population-level epidemiology. Given the lack of data on all three hypomineralisation phenotypes from most countries worldwide (Fig. 4), future research should prioritize the data acquisition from underrepresented regions to provide a more comprehensive global assessment. For these studies, participant recruitment and diagnostic criteria should ideally align with EAPD recommendations [3]. Furthermore, clinical investigations should aim to assess the entire dentition for all enamel hypomineralisation phenotypes during dental examinations, maximizing data collection with minimal additional effort.

The considerable prevalence of enamel hypomineralisation worldwide has significant implications for dental health monitoring and clinical practice. While the current findings indicate that there were no changes in MIH prevalence, these conclusions should be ascertained by data from repeated cross-sectional studies utilizing highly matched cohorts and conducted at standardized intervals. Such studies would offer valuable insights for effective MIH monitoring. Further research is warranted to determine the most efficient epidemiological surveillance methods for MIH. In the clinical setting, dental practitioners should systematically assess patients for EH presentations using recognized diagnostic indices and aim to incorporate this assessment into routine examinations. This is especially relevant for high-risk populations, where a systematic monitoring approach will enable practitioners to better track the prevalence and incidence of EH and implement timely interventions based on individual patient needs.

EH, MIH, and M + IH remain prevalent worldwide with uneven distributions across countries and continents. The present data do not support the postulated increase in MIH prevalence rates. Future research should focus on collecting data from underrepresented countries, aligning with the EAPD recommendations for MIH diagnosis, and considering all EH phenotypes in dental examinations.

## Electronic supplementary material

Below is the link to the electronic supplementary material.

Supplementary Material 1

## Data Availability

The dataset used in this study is available in the supplementary materials.
