# Supplementary material for: Epidemiological trends in enamel hypomineralisation and molar-incisor hypomineralisation: a systematic review and meta-analysis
Source: Clin Oral Investig. 2025 Jun 2;29(6):327. doi: 10.1007/s00784-025-06411-4 (PMC12130135; doi:10.1007/s00784-025-06411-4)
Supplement: Supplementary file 1 — Supplementary Material 1 [file 784_2025_6411_MOESM1_ESM.docx]

**Supplementary file**

Epidemiological Trends in Enamel Hypomineralization and Molar-Incisor Hypomineralization:

A Systematic Review and Meta-Analysis

**Contents**

- **Appendix I.** Database search queries and results.
- **Appendix II.** JBI Critical Appraisal Checklist for Studies Reporting Prevalence Data.
- **Appendix III.** Studies that were excluded due to high risk of bias.
- **Appendix IV.** Summary of the data extracted from the included studies.
- References.

**Appendix I.** Database search queries and results.

| Database | Query | Results |
| --- | --- | --- |
| PubMed | (“Molar Incisor hypomineral*” OR “Molar-Incisor-hypomineral*” OR “Demarcated opacity” OR “MIH”) AND (Prevalence OR “Epidemiological study” OR “Cross-sectional study” OR “Systematic review” OR “Meta-analysis”) | 617 |
| Embase | (("Molar Incisor hypomineral*" or "Molar-Incisor-hypomineral*" or "Demarcated opacity" or "MIH") and (Prevalence or "Epidemiological study" or "Cross-sectional study" or "Systematic review" or "Meta-analysis")).mp. | 518 |
| Hand search | The extensive hand search included the reference lists of the included studies, previously published relevant reviews, and scanned journals of interest. | 14 |
| Google Scholar | (“Molar Incisor hypomineral*” OR “Molar-Incisor-hypomineral*” OR “Demarcated opacity” OR “MIH”) AND (Prevalence OR “Epidemiological study” OR “Cross-sectional study” OR “Systematic review” OR “Meta-analysis”) | 9 |

**Appendix II.** JBI Critical Appraisal Checklist for Studies Reporting Prevalence Data.

Source: Munn Z, Moola S, Lisy K, Riitano D, Tufanaru C. Methodological guidance for systematic reviews of observational epidemiological studies reporting prevalence and incidence data. Int J Evid Based Healthc. 2015;13(3):147–153. [1]

| **Questions** | **Yes** | **No** | **Unclear** | **Not Applicable** |
| --- | --- | --- | --- | --- |
| 1. Was the sample frame appropriate to address the target population? |  |  |  |  |
| 1. Were study participants sampled in an appropriate way? |  |  |  |  |
| 1. Was the sample size adequate? |  |  |  |  |
| 1. Were the study subjects and the setting described in detail? |  |  |  |  |
| 1. Was the data analysis conducted with sufficient coverage of the identified sample? |  |  |  |  |
| 1. Were valid methods used for the identification of the condition? |  |  |  |  |
| 1. Was the condition measured in a standard, reliable way for all participants? |  |  |  |  |
| 1. Was there appropriate statistical analysis? |  |  |  |  |
| 1. Was the response rate adequate, and if not, was the low response rate managed appropriately? |  |  |  |  |

Overall appraisal: Include/ Exclude/ Seek further info.

**Appendix III.** Studies that were excluded due to high risk of bias.

| **Signaling question** | **Excluded studies** |
| --- | --- |
| 1. Was the sample frame appropriate to address the target population? (N=12) | Ibrahim et al. 2022 [2], Jan et al. 2000, Hölttä et al. et al. 2001 [3], Cots et al. et al. 2024 [4], Samec et al. et al. 2022 [5], Ngoc et al. et al. 2018 [6], Onyia et al. et al. 2023 [7], Lacerda et al. et al. 2024 [8], Lim et al. et al. 2023 [9], Ajzman et al. et al. 2023, Dybek et al. et al. 2022, Alvarado-Gaytán et al. et al. 2024 [10] |
| 2. Were study participants sampled in an appropriate way? (N=17) | Balmer et al. et al. 2005 [11], Stoica et al. et al. 2023 [12], Gomez et al. et al. 2011 [13], Hussein et al. et al. 2015 [14], Jans Munoz et al. et al. 2011 [15], Ullah et al. et al. 2016 [16], Hysi et al. et al. 2016 [17], Sidhu et al. 2020 [18], Kilinc et al. 2019 [19], Ajzman et al. 2023 [20], Lygidakis et al. 2008 [21], Allazzam et al. 2014 [22], Condo et al. 2012 [23], Ilczuk-Rypula et al. 2022 [24], Goswami et al. 2019 [25], Saber et al. 2018 [26], Zameer et al. 2024 [27] |
| 3. Was the sample size adequate? | - |
| 4. Were the study subjects and the setting described in detail? (N=2) | Kim et al. 2016 [28], Mishra et al. 2016 [29] |
| 5. Was the data analysis conducted with sufficient coverage of the identified sample? | - |
| 6. Were valid methods used for the identification of the condition? (N=5) | Khanmohammadi et al. 2022 [30], Basha et al. 2014 [31], Elfrink et al. 2012 [32], Soares et al. 2024 [33], Winkler et al. 2024 [34] |
| 7. Was the condition measured in a standard, reliable way for all participants? | - |
| 8. Was there appropriate statistical analysis? | - |
| 9. Was the response rate adequate, and if not, was the low response rate managed appropriately? | - |

**Appendix IV.** Summary of the data extracted from the included studies [35–171].

| Study ID | Year | Age  (years) | Sample size | EH prevalence | MIH prevalence | M+IH prevalence | Diagnosis index | Country |
| --- | --- | --- | --- | --- | --- | --- | --- | --- |
| Abdalla et al 2021 | 2021 | 8 to 11 | 568 |  | 20.1% | 12.5% | EAPD | Sudan |
| Abdelaziz et al 2022 | 2022 | 4 to 12 | 32142 | 7.5% | 6.6% |  | Other | Switzerland |
| Abo Elsoud et al 2019 | 2019 | 8 to 12 | 1312 |  | 13.1% |  | EAPD | Egypt |
| Afzal et al 2023 | 2023 | 7 to 9 | 3013 |  | 28.2% |  | EAPD | Norway |
| Ahmad et al 2019 | 2019 | 7 to 9 | 779 |  | 7.6% |  | EAPD | United Arab Emirates |
| Ahmadi et al 2012 | 2012 | 7 to 9 | 433 |  | 12.7% |  | DDE | Iran |
| Al-Nerabieah et al 2023 | 2023 | 8 to 11 | 1138 |  | 39.9% |  | EAPD | Syria |
| Alhowaish et al 2021 | 2021 | 8 to 10 | 893 |  | 40.5% |  | EAPD | Saudi Arabia |
| Almuallem et al 2021 | 2021 | 8 to 12 | 1562 |  | 15.2% | 12.1% | EAPD | Saudi Arabia |
| Amend et al 2021 | 2021 | 6 to 12 | 2103 |  | 13.5% |  | EAPD | Germany |
| Argote Quispe et al 2021 | 2021 | 7 to 10 | 404 |  | 19.8% |  | mDDE | Peru |
| Arheiam et al 2021 | 2021 | 8 to 10 | 1047 |  | 15.5% | 7.6% | EAPD | Libya |
| Arrow et al 2009 | 2009 | 6 to 9 | 550 | 54.7% | 21.5% |  | mDDE | Australia |
| Balmer et al 2012 | 2012 | 12 | 3233 |  | 15.9% |  | mDDE | United Kingdom |
| Bonzanini et al 2021 | 2021 | 9.5 to 13.5 | 513 |  | 19.7% |  | EAPD | Brazil |
| Brejawi et al 2023 | 2023 | 7 to 9 | 162 |  | 38.9% |  | Ghanim et al | United Arab Emirates |
| Buchgraber et al 2017 | 2017 | 6 to 12 | 1111 |  | 7.0% | 5.8% | EAPD | Austria |
| Calderara et al 2005 | 2005 | 7 to 8 | 227 | 26.9% | 13.7% | 5.8% | Other | Italy |
| Cho et al 2008 | 2008 | 11 to 14 | 2635 |  | 2.8% | 1.3% | Other | China |
| Ciocan et al 2023 | 2023 | 8 to 11 | 266 |  | 14.3% |  | EAPD | Romania |
| Da Costa-Silva et al 2010 | 2010 | 6 to 12 | 918 |  | 19.8% | 10.0% | EAPD | Brazil |
| Davenport et al 2019 | 2019 | 7 to 12 | 375 |  | 9.6% | 5.1% | EAPD | United States of America |
| Dietrich et al 2003 | 2003 | 10 to 17 | 2408 | 6.3% | 5.6% | 2.4% | mDDE | Germany |
| Dourado et al 2021 | 2021 | 8 to 14 | 246 |  | 46.6% |  | EAPD | Brazil |
| Duarte et al 2021 | 2021 | 11 to 14 | 400 |  | 18.0% |  | MIH-SSS | Brazil |
| Elzein et al 2020 | 2020 | 7 to 9 | 659 |  | 26.7% | 24.9% | Ghanim et al | Lebanon |
| Elzein et al 2021 | 2021 | 10 to 13 | 682 |  | 22.9% | 12.5% | Ghanim et al | Lebanon |
| Emmatty et al 2020 | 2020 | 8 to 15 | 5318 |  | 4.1% |  | EAPD | India |
| Estivals et al 2023 | 2023 | 7 to 9 | 856 |  | 18.7% |  | EAPD | France |
| Farias et al 2021 | 2021 | 8 to 10 | 471 |  | 9.8% | 4.5% | Ghanim et al | Brazil |
| Fernandes et al 2021(a) | 2021 | 11 to 14 | 463 |  | 10.8% | 3.5% | Ghanim et al | Brazil |
| Fernandes et al 2021(b) | 2021 | 6 to 12 | 610 |  | 9.8% |  | EAPD | Brazil |
| Figueiredo et al 2017 | 2017 | 6 to 11 | 858 |  | 14.7% |  | EAPD | Brazil |
| Folayan et al 2018 | 2018 | 6 to 16 | 853 | 7.6% | 2.9% |  | EAPD | Nigeria |
| Fteita et al 2006 | 2006 | 7 to 9 | 154 | 9.1% | 2.9% |  | mDDE | Libya |
| Gambetta-Tessini et al 2018 | 2018 | 6 to 12 | 327 | 26.9% | 14.7% |  | EAPD | Australia |
| Gambetta-Tessini et al 2019 | 2019 | 6 to 12 | 577 |  | 15.8% |  | Ghanim et al | Chile |
| Garcia-Margarit et al 2014 | 2014 | 8 to 9 | 840 |  | 21.8% | 12.4% | EAPD | Spain |
| Garcia-Perez et al 2022 | 2022 | 8 to 10 | 663 |  | 38.6% |  | EAPD | Mexico |
| Ghanim et al 2011 | 2011 | 7 to 9 | 823 |  | 18.6% | 9.0% | EAPD | Iraq |
| Ghanim et al 2014 | 2014 | 9 to 11 | 810 |  | 20.2% |  | EAPD | Iran |
| Glodkowska et al 2019 | 2019 | 6 to 12 | 1369 |  | 6.4% | 3.2% | Weerheijm | Poland |
| Glodkowska et al 2020 | 2020 | 6 to 12 | 2275 |  | 9.3% |  | EAPD | Poland |
| Grieshaber et al 2022 | 2022 | 6.5 to 18 | 1252 |  | 14.8% |  | EAPD | Switzerland |
| Groselj et al 2013 | 2013 | 6 to 11.5 | 478 |  | 21.4% | 10.4% | EAPD | Slovenia |
| Gurrusquieta et al 2017 | 2017 | 6 to 12 | 1156 |  | 15.8% |  | EAPD | Mexico |
| Hamdan et al 2020 | 2020 | 8 to 9 | 1412 |  | 13.2% |  | EAPD | Jordan |
| Harz et al 2023 | 2023 | 6 to 12 | 1270 |  | 12.8% |  | EAPD | Chile |
| Hasenauer et al 2010 | 2010 | 7 | 1283 |  | 10.9% | 2.6% | Wetzel und Reckel | Austria |
| Heitmüller et al 2013 | 2013 | 10 | 693 | 36.5% | 14.7% | 9.4% | EAPD | Germany |
| Hernandez et al 2014 | 2014 | 6 to 11 | 705 |  | 8.0% |  | EAPD | Spain |
| Hussain et al 2018 | 2018 | 8 to 12 | 342 |  | 27.2% | 9.4% | EAPD | United Arab Emirates |
| Irigoyen-Camacho et al 2020 | 2020 | 6 to 8 | 317 |  | 31.9% |  | EAPD | Mexico |
| Jälevik et al 2001 | 2001 | 7 to 8 | 516 | 33.3% | 18.4% | 8.7% | mDDE | Sweden |
| Jankovic et al 2014 | 2014 | 8 | 141 | 18.4% | 12.8% | 6.4% | EAPD | Bosnia and Herzegovina |
| Jasulaityte et al 2007 | 2007 | 7 to 9 | 1317 | 14.9% | 9.7% | 2.2% | Weerheijm | Lithuania |
| Jasulaityte et al 2008 | 2008 | 9 | 442 |  | 14.3% |  | Weerheijm | Netherlands |
| Jeremias et al 2013 | 2013 | 6 to 12 | 1157 |  | 12.3% |  | EAPD | Brazil |
| Jordi et al 2014 (a) | 2014 | 7 to 13 | 1090 |  | 16.1% |  | Mathu-Muju and Wright | Argentina |
| Jordi et al 2014 (b) | 2014 | 7 to 13 | 626 |  | 12.3% |  | Mathu-Muju and Wright | Uruguay |
| Jurlina et al 2020 | 2020 | 8 | 729 |  | 13.0% | 6.6% | EAPD | Croatia |
| Kemoli 2008 | 2008 | 6 to 8 | 3591 |  | 13.7% | 9.2% | Other | Kenya |
| Kevrekidou et al 2015 | 2015 | 8 to 14 | 2335 |  | 21.0% | 11.4% | EAPD | Greece |
| Khan et al 2022 | 2022 | 8 to 12 | 2300 |  | 4.0% | 2.7% | EAPD | India |
| Kirthiga et al 2015 | 2015 | 11 to 16 | 2000 |  | 8.9% | 2.4% | Other | India |
| Koruyucu et al 2018 | 2018 | 8 to 11 | 1511 |  | 14.2% |  | EAPD | Türkiye |
| Krishnan et al 2015 | 2015 | 9 to 14 | 4989 |  | 7.7% |  | EAPD | India |
| Kühnisch et al 2018 | 2018 | 15 | 1302 | 40.2% | 17.2% | 9.8% | EAPD | Germany |
| Kukleva et al 2008 | 2008 | 7 to 14 | 2960 |  | 3.6% |  | EAPD | Bulgaria |
| Kuscu et al 2009 | 2009 | 7 to 10 | 153 |  | 9.2% | 5.9% | EAPD | Türkiye |
| Lago et al 2022 | 2022 | 6 to 12 | 545 |  | 14.3% | 0.6% | EAPD | Brazil |
| Leppäniemi et al 2001 | 2001 | 7 to 13 | 488 |  | 19.3% |  | Alaluusua et al 1996 | Finland |
| Li et al 2012 | 2012 | 6 to 11 | 988 |  | 25.5% |  | EAPD | China |
| Lima et al 2015 | 2015 | 11 to 14 | 594 |  | 18.4% |  | EAPD | Brazil |
| Mahoney et al 2009 | 2009 | 7 to 10 | 522 | 15.3% | 14.9% | 2.9% | mDDE | New Zealand |
| Mahoney et al 2011 | 2011 | 7 to 10 | 234 |  | 18.8% | 9.0% | EAPD | New Zealand |
| Mariam et al 2021 | 2021 | 8 to 12 | 3176 |  | 11.7% |  | EAPD | India |
| Mejia et al 2019 | 2019 | 6 to 15 | 1075 |  | 11.2% |  | EAPD | Colombia |
| Mittal 2016 | 2016 | 12 to 16 | 1726 | 13.2% | 9.8% | 4.1% | EAPD | India |
| Mittal et al 2014 | 2014 | 6 to 9 | 1792 |  | 6.3% | 1.6% | EAPD | India |
| Mittal et al 2015 | 2015 | 6 to 8 | 978 |  | 7.4% |  | EAPD | India |
| Mittal et al 2016 | 2016 | 6 to 12 | 886 |  | 7.1% |  | EAPD | India |
| Mulic et al 2017 | 2017 | 8 to 9 | 103 |  | 11.7% |  | EAPD | Bosnia and Herzegovina |
| Muratbegovic et al 2007 | 2007 | 12 | 560 | 32.8% | 12.3% | 11.4% | EAPD | Bosnia and Herzegovina |
| Muratbegovic et al 2020 | 2020 | 6 to 9 | 444 |  | 11.5% | 7.4% | Weerheijm | Bosnia and Herzegovina |
| Negre-Barber et al 2018 | 2018 | 8 to 9 | 414 |  | 24.2% |  | EAPD | Spain |
| Ng et al 2015 | 2015 | 7 | 1083 |  | 12.5% | 2.8% | EAPD | Singapore |
| Nisii et al 2022 | 2022 | 7 to 8 | 346 |  | 18.2% | 5.2% | MIH-SSS | Italy |
| Olczak-Kowalczyk et al 2023 | 2023 | 18 | 1611 | 11.2% | 0.6% |  | MIH-TNI | Poland |
| Opydo-Szymaczek et al 2015 | 2015 | 6 to 14 | 470 | 25.7% | 14.9% | 12.6% | mDDE | Poland |
| Ordonez-Romero et al 2019 | 2019 | 7 to 12 | 249 |  | 9.2% |  | EAPD | Ecuador |
| Oreano et al 2023 | 2023 | 8 to 10 | 1342 |  | 7.7% |  | EAPD | Brazil |
| Ortega-Luengo et al 2024 | 2024 | 8 to 16 | 489 |  | 28.4% | 18.2% | EAPD | Spain |
| Oyedele et al 2015 | 2015 | 8 to 10 | 469 |  | 17.7% | 9.8% | EAPD | Nigeria |
| Padavala et al 2018 | 2018 | 7 to 12 | 170 |  | 12.9% | 5.3% | EAPD | India |
| Parikh et al 2012 | 2012 | 8 to 12 | 1366 |  | 9.2% | 7.6% | EAPD | India |
| Petrou et al 2014 | 2014 | 7 to 10 | 2395 |  | 10.1% | 1.2% | EAPD | Germany |
| Pitiphat et al 2014 | 2014 | 6 to 7 | 484 |  | 20.0% | 1.9% | EAPD | Thailand |
| Preusser et al 2007 | 2007 | 6 to 12 | 1002 |  | 5.9% | 3.4% | Wetzel and Reckel | Germany |
| Quintero et al 2022 | 2022 | 6 to 7 | 450 |  | 25.1% |  | Ghanim et al | Colombia |
| Rai et al 2018 | 2018 | 7 to 9 | 992 |  | 21.4% |  | mDDE | India |
| Rai et al 2019 | 2019 | 9 to 12 | 1600 |  | 13.1% | 9.2% | EAPD | India |
| Raposo et al 2019 | 2019 | 8 | 631 |  | 16.1% |  | Other | Brazil |
| Reis et al 2021 | 2021 | 8 | 450 |  | 28.7% | 12.7% | EAPD | Brazil |
| Reyes et al 2019 | 2019 | 8 | 731 |  | 12.1% | 6.6% | EAPD | Brazil |
| Rodriguez-Rodriguez et al 2021 | 2021 | 6 to 12 | 142 |  | 25.4% |  | EAPD | Venezuela |
| Saitoh et al 2018 | 2018 | 7 to 9 | 4496 |  | 19.8% |  | EAPD | Japan |
| Saldarriaga et al 2021 | 2021 | 8 to 12 | 77 |  | 14.4% |  | EAPD | Colombia |
| Salem et al 2016 | 2016 | 6 to 13 | 553 |  | 18.4% |  | EAPD | Iran |
| Schmalfuss et al 2016 | 2016 | 16 | 794 |  | 13.9% | 5.8% | EAPD | Norway |
| Shah et al 2023 | 2023 | 8 to 13 | 3000 |  | 9.5% |  | EAPD | India |
| Shin et al 2010 | 2010 | 11 | 1344 |  | 6.0% |  | Other | South Korea |
| Shrestha et al 2014 | 2014 | 7 to 12 | 747 |  | 13.7% | 11.6% | EAPD | Nepal |
| Sönmez et al 2013 | 2013 | 7 to 12 | 4018 |  | 7.7% |  | Weerheijm | Türkiye |
| Sosa-Soto et al 2022 | 2022 | 8 | 613 |  | 12.4% |  | Ghanim et al | Mexico |
| Souza et al 2013 | 2013 | 7 to 12 | 1151 |  | 12.3% |  | EAPD | Brazil |
| Soviero et al 2009 | 2009 | 7 to 13 | 249 |  | 40.2% | 18.9% | EAPD | Brazil |
| Subramaniam et al 2016 | 2016 | 7 to 9 | 2500 |  | 1.6% | 0.5% | Weerheijm | India |
| Tarannum et al 2021 | 2021 | 8 to 14 | 2250 |  | 2.1% |  | EAPD | India |
| Thakur et al 2020 | 2020 | 8 to 16 | 2000 |  | 2.9% | 2.4% | EAPD | India |
| Tourino et al 2016 | 2016 | 8 to 9 | 1181 |  | 20.4% |  | EAPD | Brazil |
| Vanhee et al 2022 | 2022 | 8 to 9.5 | 290 | 21.7% | 18.6% |  | Ghanim et al | Belgium |
| Verma et al 2022 | 2022 | 8 to 16 | 5585 |  | 7.6% |  | DDE | India |
| Vicioni-Marques et al 2023 | 2023 | 6 to 12 | 1830 |  | 16.9% |  | Ghanim et al | Brazil |
| Villanueva-Gutierrez et al 2019(b) | 2019 | 7 to 12 | 686 |  | 35.4% |  | EAPD | Mexico |
| Weerheijm et al 2001 | 2001 | 11 | 497 |  | 9.7% | 3.0% | Weerheijm | Netherlands |
| Wogelius et al 2008 | 2008 | 6 to 8 | 647 |  | 37.5% | 9.7% | EAPD | Denmark |
| Wogelius et al 2010 | 2010 | 6 to 8 | 647 |  | 37.3% |  | Weerheijm | Denmark |
| Wogelius et al 2020 | 2020 | 9 | 1837 |  | 29.5% |  | Weerheijm | Denmark |
| Wuollet et al 2014 | 2014 | 7 to 13 | 818 |  | 17.1% |  | EAPD | Finland |
| Wuollet et al 2016 | 2016 | 7 to 12 | 287 |  | 11.5% | 6.3% | EAPD | Finland |
| Yannam et al 2016 | 2016 | 8 to 12 | 2864 |  | 9.7% |  | EAPD | India |
| Yi et al 2020 | 2020 | 12 to 15 | 6523 |  | 10.0% | 2.9% | EAPD | China |
| Zagdwon et al 2002 | 2002 | 7 | 307 |  | 14.6% |  | DDE | United Kingdom |
| Zawaideh et al 2011 | 2011 | 7 to 9 | 3241 |  | 17.6% | 6.0% | EAPD | Jordan |

**References**

[1] Munn Z, MClinSc SM, Lisy K, Riitano D, Tufanaru C. Methodological guidance for systematic reviews of observational epidemiological studies reporting prevalence and cumulative incidence data. Int J Evid Based Healthc 2015;13:147–53. https://doi.org/10.1097/XEB.0000000000000054.

[2] Atef Abdelsattar Ibrahim H, Abdallah Nasr R, Adel Salama A, Ahmed Amin A. Childhood malnutrition and hypo mineralized  molar defects ;a cross sectional study, Egypt. F1000Res 2021;10. https://doi.org/10.12688/F1000RESEARCH.74557.2.

[3] Hölttä P, Kiviranta H, Leppäniemi A, Vartiainen T, Lukinmaa PL, Alaluusua S. Developmental Dental Defects in Children Who Reside by a River Polluted by Dioxins and Furans. Archives of Environmental Health: An International Journal 2001;56:522–8. https://doi.org/10.1080/00039890109602901.

[4] Cots E, Casas M, Gregoriano M, Busquet-Dura X, Bielsa J, Chacon C, et al. “Ethnic disparities in the prevalence of Molar-Incisor-Hypomineralisation (MIH) and caries among 6-12-year-old children in Catalonia, Spain.” Eur J Paediatr Dent 2024;25:1. https://doi.org/10.23804/EJPD.2024.2029.

[5] Samec T, Jan J. Developmental defects of enamel among Slovenian asthmatic children. Eur J Paediatr Dent 2022;23:121–4. https://doi.org/10.23804/EJPD.2022.23.02.14.

[6] Ngoc VTN, Huong LT, Van Nhon B, Tan NTM, Van Thuc P, Hien VTT, et al. The higher prevalence of developmental defects of enamel in the dioxin-affected region than non-dioxin-affected region: result from a cross-sectional study in Vietnam. Odontology 2019;107:17–22. https://doi.org/10.1007/S10266-018-0358-1/TABLES/4.

[7] Onyia NE, Akhigbe P, Osagie E, Obuekwe O, Omoigberale A, Richards VP, et al. Prevalence and associated factors of enamel developmental defects among Nigerian children with perinatal HIV exposure. J Clin Pediatr Dent 2023;47:1–9. https://doi.org/10.22514/JOCPD.2023.007.

[8] Wanderley Lacerda RH, Filgueiras VM, Guedes Mendonça AC, Vieira AR. Molar-incisor hypomineralization in a cohort of individuals born with cleft lip and palate. Orthod Craniofac Res 2024;27 Suppl 1:21–6. https://doi.org/10.1111/OCR.12708.

[9] Lim C, Jensen ED, Poirier BF, Sethi S, Smart G, Peña AS. Molar-incisor hypomineralisation prevalence in a cohort of Australian children with type 1 diabetes. Eur Arch Paediatr Dent 2023;24:117–23. https://doi.org/10.1007/S40368-022-00765-Z.

[10] Alvarado-Gaytán J, Saavedra-Marbán G, Velayos-Galán L, Gallardo-López NE, de Nova-García MJ, Caleya AM. Dental Developmental Defects: A Pilot Study to Examine the Prevalence and Etiology in a Population of Children between 2 and 15 Years of Age. Dentistry Journal 2024, Vol 12, Page 84 2024;12:84. https://doi.org/10.3390/DJ12040084.

[11] Balmer R, Toumba KJ, Munyombwe T, Duggal MS. A comparison of the presentation of molar incisor hypomineralisation in two communities with different fluoride exposure. European Archives of Paediatric Dentistry 2015;16:257–64. https://doi.org/10.1007/s40368-014-0170-8.

[12] Stoica SN, Nimigean V, Moraru SA, Sîrbu I, Nimigean VR. A clinical and statistical study on enamel hypomineralization of the first permanent molar in the period of mixed dentition. Rom J Morphol Embryol 2023;64:241–9. https://doi.org/10.47162/RJME.64.2.15.

[13] Martínez Gómez TP, Guinot Jimeno F, Bellet Dalmau LJ, Giner Tarrida L. Prevalence of molar-incisor hypomineralisation observed using transillumination in a group of children from Barcelona (Spain). Int J Paediatr Dent 2012;22:100–9. https://doi.org/10.1111/J.1365-263X.2011.01172.X.

[14] Hussein AS, Faisal M, Haron M, Ghanim AM, Abu-Hassan MI. Distribution of Molar Incisor Hypomineralization in Malaysian Children Attending University Dental Clinic. J Clin Pediatr Dent 2015;39:219–23. https://doi.org/10.17796/1053-4628-39.3.219.

[15] Jans Muñoz A, Díaz Meléndez J, Vergara González C, Zaror Sánchez C. Frequency and Severity of the Molar Incisor Hypomineralization in Patients Treated at the Dental Clinic of the Universidad de La Frontera, Chile. International Journal of Odontostomatology 2011;5:133–40. https://doi.org/10.4067/S0718-381X2011000200004.

[16] Ullah I, Parveen N, Shabbir R. Pattern and Presentaion of Molar Incisor Hypomineralizaion in Pakistani Children. International Journal of Contemporary Medical Research 2016;3.

[17] Hysi D, Kuscu OO, Droboniku E, Toti C, Xhemnica L, Caglar E. Prevalence and aetiology of Molar-Incisor Hypomineralisation among children aged 8-10 years in Tirana, Albania. Eur J Paediatr Dent 2016;17:75–9.

[18] Sidhu N, Wang Y, Barrett E, Casas M. Prevalence and presentation patterns of enamel hypomineralisation (MIH and HSPM) among paediatric hospital dental patients in Toronto, Canada: a cross-sectional study. European Archives of Paediatric Dentistry 2020;21:263–70. https://doi.org/10.1007/S40368-019-00477-X/FIGURES/5.

[19] Kılınç G, Çetin M, Köse B, Ellidokuz H. Prevalence, aetiology, and treatment of molar incisor hypomineralization in children living in Izmir City (Turkey). Int J Paediatr Dent 2019;29:775–82. https://doi.org/10.1111/IPD.12508.

[20] Berenstein Ajzman G, Dagon N, Iraqi R, Blumer S, Fadela S. The Prevalence of Developmental Enamel Defects in Israeli Children and Its Association with Perinatal Conditions: A Cross-Sectional Study. Children 2023, Vol 10, Page 903 2023;10:903. https://doi.org/10.3390/CHILDREN10050903.

[21] Lygidakis NA, Dimou G, Briseniou E. Molar-incisor-hypomineralisation (MIH). Retrospective clinical study in Greek children. I. Prevalence and defect characteristics. Eur Arch Paediatr Dent 2008;9:200–6. https://doi.org/10.1007/BF03262636.

[22] Allazzam SM, Alaki SM, El Meligy OAS. Molar Incisor Hypomineralization, Prevalence, and Etiology. Int J Dent 2014;2014:234508. https://doi.org/10.1155/2014/234508.

[23] Condò R, Perugia C, Maturo P, Docimo R. MIH: epidemiologic clinic study in paediatric patient. Oral Implantol (Rome) 2012;5:58.

[24] Ilczuk-Rypuła D, Zalewska M, Pietraszewska D, Dybek A, Nitecka-Buchta A, Postek-Stefańska L. Prevalence and Possible Etiological Factors of Molar-Incisor Hypomineralization (MIH) in Population of Silesian Children in Poland: A Pilot Retrospective Cohort Study. International Journal of Environmental Research and Public Health 2022, Vol 19, Page 8697 2022;19:8697. https://doi.org/10.3390/IJERPH19148697.

[25] Goswami M, Bhushan U, Pandiyan R, Sharma S. Molar Incisor Hypomineralization—An Emerging Burden: A Short Study on Prevalence and Clinical Characteristics in Central Delhi, India. Int J Clin Pediatr Dent 2019;12:211. https://doi.org/10.5005/JP-JOURNALS-10005-1624.

[26] Saber F, Waly N, Moheb D. Prevalence of molar incisor hypomineralisation in a group of Egyptian children using the short form: a cross-sectional study. European Archives of Paediatric Dentistry 2018;19:337–45. https://doi.org/10.1007/S40368-018-0364-6/FIGURES/5.

[27] Zameer M, Wali Peeran S, Nahid Basheer S, Ali Peeran S, Anwar Naviwala G, Badiujjama Birajdar S. Molar incisor hypomineralization: Prevalence, severity and associated aetiological factors in children seeking dental care at Armed Forces Hospital Jazan, Saudi Arabia. Saudi Dent J 2024. https://doi.org/10.1016/J.SDENTJ.2024.06.003.

[28] Kim T, Jeong I, Lee D, Kim J, Yang Y. Prevalence and Etiology of Molar Incisor Hypomineralization in Children Aged 8 - 9 Years. Journal of the Korean Academy of Pediatric Dentistry 2016;43:410–8. https://doi.org/10.5933/JKAPD.2016.43.4.410.

[29] Mishra A, Pandey RK. Molar Incisor Hypomineralization: An Epidemiological Study with Prevalence and Etiological Factors in Indian Pediatric Population. Int J Clin Pediatr Dent 2016;9:167–71. https://doi.org/10.5005/JP-JOURNALS-10005-1357.

[30] Khanmohammadi R, Seraj B, Salari A, Alipour F. Etiological Factors Involved in Molar-Incisor Hypomineralization in 7 to 12-Year-Old Children in Tehran. Front Dent 2022;19. https://doi.org/10.18502/FID.V19I16.9962.

[31] Basha S, Mohamed RN, Swamy HS. Prevalence and associated factors to developmental defects of enamel in primary and permanent dentition - PubMed. Oral Health Dental Management 2014;13:588–94.

[32] Elfrink MEC, Ghanim A, Manton DJ, Weerheijm KL. Standardised studies on Molar Incisor Hypomineralisation (MIH) and Hypomineralised Second Primary Molars (HSPM): a need. European Archives of Paediatric Dentistry 2015;16:247–55. https://doi.org/10.1007/s40368-015-0179-7.

[33] Soares LS, Fernandes EC, Santos PB. The prevalence and characteristics of molar-incisor hypomineralisation in Natal, Brazil. Pediatric Dental Journal 2024;34:14–8. https://doi.org/10.1016/J.PDJ.2023.12.003.

[34] Winkler JR, Dixon BL, Singh I, Soto R, Qiu Y, Zhang Y, et al. Prenatal exposure to environmental toxins and comprehensive dental findings in a population cohort of children. BMC Oral Health 2024;24:1–8. https://doi.org/10.1186/S12903-023-03786-2/FIGURES/2.

[35] Haque Afzal S, Wigen TI, Skaare AB, Brusevold IJ. Molar-incisor hypomineralisation in Norwegian children: Prevalence and associated factors. Eur J Oral Sci 2023;131. https://doi.org/10.1111/EOS.12930.

[36] Dourado DG, Lima CCB, Silva RNC, Tajra FS, Moura MS, Lopes TSP, et al. Molar-incisor hypomineralization in quilombola children and adolescents: A study of prevalence and associated factors. J Public Health Dent 2021;81:178–87. https://doi.org/10.1111/JPHD.12429.

[37] Grieshaber A, Waltimo T, Haschemi AA, Erb J, Steffen R, Bornstein MM, et al. Prevalence of and factors associated with molar-incisor hypomineralisation in schoolchildren in the canton of Basel-Landschaft, Switzerland. Clin Oral Investig 2023;27:871–7. https://doi.org/10.1007/S00784-022-04648-X.

[38] Harz D, Catalán Gamonal B, Matute García S, Jeremias F, Martin J, Fresno MC. Prevalence and severity of molar-incisor hypomineralization, is there an association with socioeconomic status? A cross-sectional study in Chilean schoolchildren. European Archives of Paediatric Dentistry 2023;24:577–84. https://doi.org/10.1007/S40368-023-00820-3/FIGURES/4.

[39] Brejawi M, Venkiteswaran A, Ergieg SMO, Md Sabri BA. Caries experience in children with molar–incisor hypomineralisation in Fujairah, United Arab Emirates and its association with hypomineralised teeth number. European Archives of Paediatric Dentistry 2024;25:211–6. https://doi.org/10.1007/S40368-024-00867-W/TABLES/2.

[40] Bonzanini LIL, Da Arduim AS, Lenzi TL, Hugo FN, Hilgert JB, Casagrande L. Molar-incisor hypomineralization and dental caries: A hierarchical approach in a populational-based study. Braz Dent J 2021;32:74–82. https://doi.org/10.1590/0103-6440202104511.

[41] Balmer R, Toumba J, Godson J, Duggal M. The prevalence of molar incisor hypomineralisation in Northern England and its relationship to socioeconomic status and water fluoridation. Int J Paediatr Dent 2012;22:250–7. https://doi.org/10.1111/J.1365-263X.2011.01189.X.

[42] Arrow P. Risk factors in the occurrence of enamel defects of the first permanent molars among schoolchildren in Western Australia. Community Dent Oral Epidemiol 2009;37:405–15. https://doi.org/10.1111/J.1600-0528.2009.00480.X.

[43] Arheiam A, Abbas S, Ballo L, Borowis E, Rashwan S, El Tantawi M. Prevalence, distribution, characteristics and associated factors of molar-incisor hypo-mineralisation among Libyan schoolchildren: a cross-sectional survey. European Archives of Paediatric Dentistry 2021;22:595–601. https://doi.org/10.1007/S40368-020-00594-Y/TABLES/2.

[44] Quispe DMA, De Priego GPM, Manco RAL, Portaro CP. Molar incisor hypomineralization: Prevalence and severity in schoolchildren of Puno, Peru. J Indian Soc Pedod Prev Dent 2021;39:246–50. https://doi.org/10.4103/JISPPD.JISPPD_460_20.

[45] Amend S, Nossol C, Bausback-Schomakers S, Wleklinski C, Scheibelhut C, Pons-Kühnemann J, et al. Prevalence of molar-incisor-hypomineralisation (MIH) among 6–12-year-old children in Central Hesse (Germany). Clin Oral Investig 2021;25:2093–100. https://doi.org/10.1007/S00784-020-03519-7/TABLES/5.

[46] Almuallem Z, Alsuhaim A, Alqudayri A, Aljarid S, Mousa Alotaibi M, Alkraida R, et al. Prevalence and possible aetiological factors of molar incisor hypomineralisation in Saudi children: A cross-sectional study. Saudi Dent J 2022;34:36–44. https://doi.org/10.1016/J.SDENTJ.2021.10.004.

[47] Alhowaish L, Baidas L, Aldhubaiban M, Bello LL, Al-Hammad N. Etiology of Molar-Incisor Hypomineralization (MIH): A Cross-Sectional Study of Saudi Children. Children 2021, Vol 8, Page 466 2021;8:466. https://doi.org/10.3390/CHILDREN8060466.

[48] Al-Nerabieah Z, AlKhouli M, Dashash M. Prevalence and clinical characteristics of molar-incisor hypomineralization in Syrian children: a cross-sectional study. Scientific Reports 2023 13:1 2023;13:1–8. https://doi.org/10.1038/s41598-023-35881-3.

[49] Ahmad SH, Petrou MA, Alhumrani A, Hashim R, Splieth C. Prevalence of Molar-Incisor Hypomineralisation in an Emerging Community, and a Possible Correlation with Caries, Fluorosis and Socioeconomic Status. Oral Health Prev Dent 2019;17:323–7. https://doi.org/10.3290/J.OHPD.A42725.

[50] Abo ElSoud AA, Mahfouz SM. Prevalence and severity of Molar Incisor Hypomineralization in School Children of Suez Canal Region: Cross-Sectional Study. Egypt Dent J 2019;65:909–15. https://doi.org/10.21608/EDJ.2015.71986.

[51] Abdelaziz M, Krejci I, Banon J. Prevalence of Molar Incisor Hypomineralization in over 30,000 Schoolchildren in Switzerland. J Clin Pediatr Dent 2022;46:1–5. https://doi.org/10.17796/1053-4625-46.1.1.

[52] Abdalla HE, Abuaffan AH, Kemoli AM. Molar incisor hypomineralization, prevalence, pattern and distribution in Sudanese children. BMC Oral Health 2021;21:1–8. https://doi.org/10.1186/S12903-020-01383-1/TABLES/4.

[53] Ahmadi R, Ramazani N, Nourinasab R. Molar incisor hypomineralization: a study of prevalence and etiology in a group of Iranian children - PubMed. Iran J Pediatrics 2012;22:245–51.

[54] Dietrich G, Sperling S, Hetzer G. Molar incisor hypomineralisation in a group of children and adolescents living in Dresden (Germany). Eur J Paediatr Dent 2003;4:133–7.

[55] Davenport M, Welles AD, Angelopoulou M V., Gonzalez C, Okunseri C, Barbeau L, et al. Prevalence of molar-incisor hypomineralization in Milwaukee, Wisconsin, USA: a pilot study. Clin Cosmet Investig Dent 2019;11:109–17. https://doi.org/10.2147/CCIDE.S172736.

[56] da Costa-Silva CM, Jeremias F, de Souza JF, Cordeiro R de CL, Santos-Pinto L, Zuanon ACC. Molar incisor hypomineralization: prevalence, severity and clinical consequences in Brazilian children. Int J Paediatr Dent 2010;20:426–34. https://doi.org/10.1111/J.1365-263X.2010.01097.X.

[57] Cho SY, Ki Y, Chu V. Molar incisor hypomineralization in Hong Kong Chinese children. Int J Paediatr Dent 2008;18:348–52. https://doi.org/10.1111/J.1365-263X.2008.00927.X.

[58] Buchgraber B, Kqiku L, Ebeleseder KA. Molar incisor hypomineralization: proportion and severity in primary public school children in Graz, Austria. Clin Oral Investig 2018;22:757–62. https://doi.org/10.1007/S00784-017-2150-Y/TABLES/3.

[59] Calderara PC, Gerthoux PM, Mocarelli P, Lukinmaa PL, Tramacere PL, Alaluusua S. The prevalence of Molar Incisor Hypomineralisation (MIH) in a group of Italian school children. Eur J Paediatr Dent 2005;6:79–83.

[60] Ciocan B, Săndulescu M, Luca R. Real-World Evidence on the Prevalence of Molar Incisor Hypomineralization in School Children from Bucharest, Romania. Children 2023;10. https://doi.org/10.3390/CHILDREN10091563.

[61] Hamdan M, Abu-Ghefreh EA, Al-Abdallah M, Rajab LD. The prevalence and severity of molar incisor hypomineralization (MIH) among 8 year-old children in Amman, Jordan. Egypt Dent J 2020;66:1989–97. https://doi.org/10.21608/EDJ.2020.35468.1182.

[62] Gurrusquieta B, Núñez V, López M. Prevalence of molar incisor hypomineralization in Mexican children. Journal of Clinical Pediatric Dentistry 2017;14:1.

[63] Grošelj M, Jan J. Molar incisor hypomineralisation and dental caries among children in Slovenia - PubMed. Eur J Paediatr Dent 2013;14:241–5.

[64] Głódkowska N, Emerich K. The impact of environmental air pollution on the prevalence of molar incisor hypomineralization in schoolchildren: A cross-sectional study. Adv Clin Exp Med 2020;29:1469–77. https://doi.org/10.17219/ACEM/128227.

[65] Glodkowska N, Emerich K. Molar Incisor Hypomineralization: prevalence and severity among children from Nothern Poland. Eur J Paediatr Dent 2019;20:59–66. https://doi.org/10.23804/EJPD.2019.20.01.12.

[66] Ghanim A, Bagheri R, Golkari A, Manton D. Molar-incisor hypomineralisation: a prevalence study amongst primary schoolchildren of Shiraz, Iran. Eur Arch Paediatr Dent 2014;15:75–82. https://doi.org/10.1007/S40368-013-0067-Y.

[67] Ghanim A, Morgan M, Mariño R, Bailey D, Manton D. Molar-incisor hypomineralisation: prevalence and defect characteristics in Iraqi children. Int J Paediatr Dent 2011;21:413–21. https://doi.org/10.1111/J.1365-263X.2011.01143.X.

[68] García-Pérez A, Pineda ÁEGA, Gutiérrez TV, Pérez NGP, Gómez-Clavel JF. Impact of diseases of the hard tissues of teeth on oral health-related quality of life of schoolchildren in area with a high concentration of fluoride in drinking water. Community Dent Health 2022;39:240–6. https://doi.org/10.1922/CDH_00078GARCIA-PEREZ07.

[69] Garcia-Margarit M, Catalá-Pizarro M, Montiel-Company JM, Almerich-Silla JM. Epidemiologic study of molar-incisor hypomineralization in 8-year-old Spanish children. Int J Paediatr Dent 2014;24:14–22. https://doi.org/10.1111/IPD.12020.

[70] Gambetta-Tessini K, Mariño R, Ghanim A, Calache H, Manton DJ. The impact of MIH/HSPM on the carious lesion severity of schoolchildren from Talca, Chile. European Archives of Paediatric Dentistry 2019;20:417–23. https://doi.org/10.1007/S40368-019-00416-W/TABLES/3.

[71] Gambetta-Tessini K, Mariño R, Ghanim A, Calache H, Manton DJ. Carious lesion severity and demarcated hypomineralized lesions of tooth enamel in schoolchildren from Melbourne, Australia. Aust Dent J 2018;63:365–73. https://doi.org/10.1111/ADJ.12626.

[72] Fteita D, Ali A, Alaluusua S. Molar-incisor hypomineralization (MIH) in a group of school-aged children in Benghazi, Libya. Eur Arch Paediatr Dent 2006;7:92–5. https://doi.org/10.1007/BF03320821.

[73] Folayan MO, Chukwumah NM, Popoola BO, Temilola DO, Onyejaka NK, Oyedele TA, et al. Developmental defects of the enamel and its impact on the oral health quality of life of children resident in Southwest Nigeria. BMC Oral Health 2018;18:1–10. https://doi.org/10.1186/S12903-018-0622-3/TABLES/5.

[74] Fernandes IC, Forte FDS, Sampaio FC. Molar-incisor hypomineralization (MIH), dental fluorosis, and caries in rural areas with different fluoride levels in the drinking water. Int J Paediatr Dent 2021;31:475–82. https://doi.org/10.1111/IPD.12728.

[75] Freitas Fernandes LH, Laureano ICC, Farias L, Andrade NM, Soares Forte FD, Barros Alencar CR, et al. Incisor Molar Hypomineralization and Quality of Life: A Population-Based Study with Brazilian Schoolchildren. Int J Dent 2021;2021:6655771. https://doi.org/10.1155/2021/6655771.

[76] Farias L, Laureano ICC, Fernandes LHF, Forte FDS, vargas-ferreira F, de Alencar CRB, et al. Presence of molar-incisor hypomineralization is associated with dental caries in Brazilian schoolchildren. Braz Oral Res 2021;35:e13. https://doi.org/10.1590/1807-3107BOR-2021.VOL35.0013.

[77] Estivals J, Fahd C, Baillet J, Rouas P, Manton DJ, Garot E. The prevalence and characteristics of and the association between MIH and HSPM in South-Western France. Int J Paediatr Dent 2023;33:298–304. https://doi.org/10.1111/IPD.13040.

[78] Emmatty T, Eby A, Joseph M, Bijimole J, Kavita K, Asif I. The prevalence of molar incisor hypomineralization of school children in and around Muvattupuzha, Kerala. J Indian Soc Pedod Prev Dent 2020;38:14–9. https://doi.org/10.4103/JISPPD.JISPPD_152_18.

[79] Elzein R, Chouery E, Abdel-Sater F, Bacho R, Ayoub F. Relation between molar-incisor hypomineralization (MIH) occurrence and war pollutants in bombarded regions: Epidemiological pilot study in Lebanon. Niger J Clin Pract 2021;24:1808–13. https://doi.org/10.4103/NJCP.NJCP_702_20.

[80] Elzein R, Chouery E, Abdel-Sater F, Bacho R, Ayoub F. Molar incisor hypomineralisation in Lebanon: prevalence and clinical characteristics. European Archives of Paediatric Dentistry 2020;21:609–16. https://doi.org/10.1007/S40368-019-00505-W/FIGURES/3.

[81] Duarte MBS, Carvalho VR, Hilgert LA, Ribeiro APD, Leal SC, Takeshita EM. Is there an association between dental caries, fluorosis, and molar-incisor hypomineralization? Journal of Applied Oral Science 2021;29:e20200890. https://doi.org/10.1590/1678-7757-2020-0890.

[82] Figueiredo APDR, Ribeiro APD, Dos Santos-Pinto LAM, Cordeiro R de CL, Cabral RN, Leal SC. Are Hypomineralized Primary Molars and Canines Associated with Molar-Incisor Hypomineralization? - PubMed. Pediatr Dent 2017;39:445–9.

[83] Lima M de DM de, Andrade MJB, Dantas-Neta NB, Andrade NS, Teixeira RJB, Moura MS de, et al. Epidemiologic Study of Molar-incisor Hypomineralization in Schoolchildren in North-eastern Brazil - PubMed. Pediatr Dent 2015;37:513–9.

[84] Leppäniemi A, Lukinmaa PL, Alaluusua S. Nonfluoride hypomineralizations in the permanent first molars and their impact on the treatment need. Caries Res 2001;35:36–40. https://doi.org/10.1159/000047428.

[85] Li L, Li J. Investigation of molar-incisor hypomineralization among children from 6 to 11 years in Lucheng district, Wenzhou city. Shanghai Kou Qiang Yi Xue 2012;21:576–9.

[86] Damares Lago J, Restrepo M, Girotto Bussaneli D, Patrícia Cavalheiro J, Feltrin De Souza J, Santos-Pinto L, et al. Molar-Incisor Hypomineralization: Prevalence Comparative Study in 6 Years of Interval. The Scientific World Journal 2022;2022:4743252. https://doi.org/10.1155/2022/4743252.

[87] Kukleva M, Petrova S, Kondeva V, Nihtyanova TI. Molar incisor hypomineralisation in 7-to-14-year old children in Plovdiv, Bulgaria--an epidemiologic study. Folia Med (Plovdiv) 2008.

[88] Kuscu OO, Çaglar E, Aslan S, Durmusoglu E, Karademir A, Sandalli N. The prevalence of molar incisor hypomineralization (MIH) in a group of children in a highly polluted urban region and a windfarm-green energy island. Int J Paediatr Dent 2009;19:176–85. https://doi.org/10.1111/J.1365-263X.2008.00945.X.

[89] Kühnisch J, Kabary L, Malyk Y, Rothmaier K, Metz I, Hickel R, et al. Relationship between caries experience and demarcated hypomineralised lesions (including MIH) in the permanent dentition of 15-year-olds. Clin Oral Investig 2018;22:2013–9. https://doi.org/10.1007/S00784-017-2299-4/FIGURES/1.

[90] Krishnan R, Ramesh M, Chalakkal P. Prevalence and characteristics of MIH in school children residing in an endemic fluorosis area of India: an epidemiological study. Eur Arch Paediatr Dent 2015;16:455–60. https://doi.org/10.1007/S40368-015-0194-8.

[91] Koruyucu M, Özel S, Tuna EB. Prevalence and etiology of molar-incisor hypomineralization (MIH) in the city of Istanbul. J Dent Sci 2018;13:318. https://doi.org/10.1016/J.JDS.2018.05.002.

[92] Kirthiga M, Poornima P, Praveen R, Gayathri P, Manju M, Priya M. Prevalence and severity of molar incisor hypomineralization in children aged 11-16 years of a city in Karnataka, Davangere. J Indian Soc Pedod Prev Dent 2015;33:213–7. https://doi.org/10.4103/0970-4388.160366.

[93] Kevrekidou A, Kosma I, Konstantinos A, Nikolaos K. Molar Incisor Hypomineralization of Eight- and 14-year-old Children: Prevalence, Severity, and Defect Characteristics . Pediatr Dent 2015;37:455–61.

[94] Khan A, Garg N, Mayall SS, Pathivada L, Kaur H, Yeluri R. Prevalence, Pattern, and Severity of Molar Incisor Hypomineralization in 8-12-year-old Schoolchildren of Moradabad City. Int J Clin Pediatr Dent 2022;15:168–74. https://doi.org/10.5005/JP-JOURNALS-10005-2362.

[95] Kemoli AM. Prevalence Of Molar Incisor Hypomineralisation In Six To Eight Year-Olds In Two Rural Divisions In Kenya. East Afr Med J 2008;85:514–20. https://doi.org/10.4314/EAMJ.V85I10.9668.

[96] Jurlina D, Uzarevic Z, Ivanisevic Z, Matijevic N, Matijevic M. Prevalence of Molar–Incisor Hypomineralization and Caries in Eight-Year-Old Children in Croatia. International Journal of Environmental Research and Public Health 2020, Vol 17, Page 6358 2020;17:6358. https://doi.org/10.3390/IJERPH17176358.

[97] Jeremias F, Souza JF De, Costa Silva CM Da, Cordeiro RDCL, Zuanon ÂCC, Santos-Pinto L. Dental caries experience and Molar-Incisor Hypomineralization. Acta Odontol Scand 2013;71:870–6. https://doi.org/10.3109/00016357.2012.734412.

[98] López Jordi M del C, Cortese SG, Álvarez L, Salveraglio I, Ortolani AM, Biondi AM. Comparison of the prevalence of molar incisor hypomineralization among children with different health care coverage in the cities of Buenos Aires (Argentina) and montevideo (Uruguay). Salud Colect 2014;10:243–51. https://doi.org/10.18294/SC.2014.225.

[99] Jasulaityte L, Weerheijm KL, Veerkamp JS. Prevalence of molar-incisor-hypomineralisation among children participating in the Dutch National Epidemiological Survey (2003). Eur Arch Paediatr Dent 2008;9:218–23. https://doi.org/10.1007/BF03262638.

[100] Jasulaityte L, Veerkamp JS, Weerheijm KL. Molar incisor hypomineralization: review and prevalence data from the study of primary school children in Kaunas/Lithuania. Eur Arch Paediatr Dent 2007;8:87–94. https://doi.org/10.1007/BF03262575.

[101] Janković S, Ivanović M, Davidović B, Lečić J. Distribution and characteristics of molar-incisor hypomineralization. Vojnosanit Pregl 2014;71:730–4. https://doi.org/10.2298/VSP1408730J.

[102] Jälevik B, Klingberg G, Barregård L, Norén JG. The prevalence of demarcated opacities in permanent first molars in a group of Swedish children. Acta Odontol Scand 2001;59:255–60. https://doi.org/10.1080/000163501750541093.

[103] Irigoyen-Camacho ME, Villanueva-Gutierrez T, Castano-Seiquer A, Molina-Frechero N, Zepeda-Zepeda M, Sánchez-Pérez L. Evaluating the changes in molar incisor hypomineralization prevalence: A comparison of two cross-sectional studies in two elementary schools in Mexico City between 2008 and 2017. Clin Exp Dent Res 2020;6:82–9. https://doi.org/10.1002/CRE2.252.

[104] Hussein AS, Faisal M, Haron M, Ghanim AM, Abu-Hassan MI. Distribution of molar incisor hypomineralization in Malaysian children Attending University Dental Clinic. Journal of Clinical Pediatric Dentistry 2015;39:219–23. https://doi.org/10.17796/1053-4628-39.3.219.

[105] Hernández Juyol M, Muñoz S, López F, Boj Quesada JR, Espasa Suárez de Deza JE. Prevalencia de la hipomineralización incisivo molar en una muestra de 772 escolares de la provincia de Barcelona. Articles Publicats En Revistes (Odontoestomatologia) 2020;22:115–25.

[106] Heitmüller D, Thiering E, Hoffmann U, Heinrich J, Manton D, Kühnisch J, et al. Is there a positive relationship between molar incisor hypomineralisations and the presence of dental caries? Int J Paediatr Dent 2013;23:116–24. https://doi.org/10.1111/J.1365-263X.2012.01233.X.

[107] Hasenauer L, Vogelsberger M, Bürkle V, Grunert I, Meißner N. Prävalenz und Ausprägung der Molar Incisor Hypomineralisation (MIH) in Salzburg und Tirol und ein Beitrag zur Erforschung der Ursachen. Stomatologie 2010 107:3 2010;107:43–50. https://doi.org/10.1007/S00715-010-0118-5.

[108] Mittal N, Sharma BB. Hypomineralised second primary molars: prevalence, defect characteristics and possible association with Molar Incisor Hypomineralisation in Indian children. European Archives of Paediatric Dentistry 2015;16:441–7. https://doi.org/10.1007/s40368-015-0190-z.

[109] Quintero Y, Restrepo M, Rojas-Gualdrón DF, de Farias AL, Santos-Pinto L. Association between hypomineralization of deciduous and molar incisor hypomineralization and dental caries. Braz Dent J 2022;33:113–9. https://doi.org/10.1590/0103-6440202204807.

[110] Preusser SE, Ferring V, Wleklinski C, Wetzel WE. Prevalence and severity of molar incisor hypomineralization in a region of Germany -- a brief communication. J Public Health Dent 2007;67:148–50. https://doi.org/10.1111/J.1752-7325.2007.00040.X.

[111] Pitiphat W, Luangchaichaweng S, Pungchanchaikul P, Angwaravong O, Chansamak N. Factors associated with molar incisor hypomineralization in Thai children. Eur J Oral Sci 2014;122:265–70. https://doi.org/10.1111/EOS.12136.

[112] Petrou MA, Giraki M, Bissar AR, Basner R, Wempe C, Altarabulsi MB, et al. Prevalence of Molar-Incisor-Hypomineralisation among school children in four German cities. Int J Paediatr Dent 2014;24:434–40. https://doi.org/10.1111/IPD.12089.

[113] Parikh DR, Ganesh M, Bhaskar V. Prevalence and characteristics of Molar Incisor Hypomineralisation (MIH) in the child population residing in Gandhinagar, Gujarat, India. Eur Arch Paediatr Dent 2012;13:21–6. https://doi.org/10.1007/BF03262836.

[114] Padavala S, Sukumaran G. Molar Incisor Hypomineralization and Its Prevalence. Contemp Clin Dent 2018;9:S246–50. https://doi.org/10.4103/CCD.CCD_161_18.

[115] Oyedele TA, Folayan MO, Adekoya-Sofowora CA, Oziegbe EO, Esan TA. Prevalence, pattern and severity of molar incisor hypomineralisation in 8- to 10-year-old school children in Ile-Ife, Nigeria. Eur Arch Paediatr Dent 2015;16:277–82. https://doi.org/10.1007/S40368-015-0175-Y.

[116] Owlia F, Akhavan-Karbassi M-H, Rahimi R. Could Molar-Incisor Hypomineralization (MIH) Existence be Predictor of Short Stature? Int J Prev Med 2020;11:101. https://doi.org/10.4103/IJPVM.IJPVM_459_18.

[117] Ortega-Luengo S, Feijóo-Garcia G, Miegimolle-Herrero M, Gallardo-López NE, Caleya-Zambrano AM. Prevalence and clinical presentation of molar incisor hypomineralisation among a population of children in the community of Madrid. BMC Oral Health 2024;24:1–9. https://doi.org/10.1186/S12903-024-04003-4/FIGURES/3.

[118] Oreano MD avila, Santos PS, Borgatto AF, Bolan M, Cardoso M. Association between dental caries and molar-incisor hypomineralisation in first permanent molars: A hierarchical model. Community Dent Oral Epidemiol 2023;51:436–42. https://doi.org/10.1111/CDOE.12778.

[119] Ordonez-Romero I, Jijon-Granja Y, Ubilla-Mazzini W, Porro-Porro L, Alvarez-Giler G. Distribution of Molar Incisor Hypomineralization in Ecuadorian Children. Dent Hypotheses 2019;10:65–9. https://doi.org/10.4103/DENTHYP.DENTHYP_26_19.

[120] Opydo-Szymaczek J, Gerreth K. Developmental Enamel Defects of the Permanent First Molars and Incisors and Their Association with Dental Caries in the Region of Wielkopolska, Western Poland. Oral Health Prev Dent 2015;13:461–9. https://doi.org/10.3290/J.OHPD.A33088.

[121] Olczak-Kowalczyk D, Krämer N, Gozdowski D, Turska-Szybka A. Developmental enamel defects and their relationship with caries in adolescents aged 18 years. Scientific Reports 2023 13:1 2023;13:1–9. https://doi.org/10.1038/s41598-023-31717-2.

[122] Nisii F, Mazur M, De Nuccio C, Martucci C, Spuntarelli M, Labozzetta S, et al. Prevalence of molar incisor hypomineralization among school children in Rome, Italy. Sci Rep 2022;12. https://doi.org/10.1038/S41598-022-10050-0.

[123] Ng JJ, Eu OC, Nair R, Hong CHL. Prevalence of molar incisor hypomineralization (MIH) in Singaporean children. Int J Paediatr Dent 2015;25:73–8. https://doi.org/10.1111/IPD.12100.

[124] Negre-Barber A, Montiel-Company JM, Catalá-Pizarro M, Almerich-Silla JM. Degree of severity of molar incisor hypomineralization and its relation to dental caries. Scientific Reports 2018 8:1 2018;8:1–7. https://doi.org/10.1038/s41598-018-19821-0.

[125] Arslanagic-Muratbegovic A, Markovic N, Zukanovic A, Tiro A, Dzemidzic V. Molar Incisor Hypomineralization: Prevalence and severity in six to nine-year-old Sarajevo children. Eur J Paediatr Dent 2020;21:243–7. https://doi.org/10.23804/EJPD.2020.21.03.16.

[126] Muratbegovic A, Markovic N, Ganibegovic Selimovic M. Molar incisor hypomineralisation in Bosnia and Herzegovina: aetiology and clinical consequences in medium caries activity population. Eur Arch Paediatr Dent 2007;8:189–94. https://doi.org/10.1007/BF03262595.

[127] Mulic A, Cehajic E, Tveit AB, Stenhagen KR. How serious is Molar Incisor Hypomineralisation (MIH) among 8- and 9-year-old children in Bosnia-Herzegovina? A clinical study. Eur J Paediatr Dent 2017;18:153–7. https://doi.org/10.23804/EJPD.2017.18.02.12.

[128] Mittal R, Chandak S, Chandwani M, Singh P, Pimpale J. Assessment of association between molar incisor hypomineralization and hypomineralized second primary molar. J Int Soc Prev Community Dent 2016;6:34. https://doi.org/10.4103/2231-0762.175409.

[129] Mittal NP, Goyal A, Gauba K, Kapur A. Molar incisor hypomineralisation: prevalence and clinical presentation in school children of the northern region of India. Eur Arch Paediatr Dent 2014;15:11–8. https://doi.org/10.1007/S40368-013-0045-4.

[130] Mittal N. Phenotypes of Enamel Hypomineralization and Molar Incisor Hypomineralization in Permanent Dentition: Identification, Quantification and Proposal for Classification. J Clin Pediatr Dent 2016;40:367–74. https://doi.org/10.17796/1053-4628-40.5.367.

[131] Mejía JD, Restrepo M, González S, Álvarez LG, Santos-Pinto L, Escobar A. Molar Incisor Hypomineralization in Colombia: Prevalence, Severity and Associated Risk Factors. J Clin Pediatr Dent 2019;43:185–9. https://doi.org/10.17796/1053-4625-43.3.7.

[132] Mariam S, Goyal A, Dhareula A, Gauba K, Bhatia SK, Kapur A. A case–controlled investigation of risk factors associated with molar incisor hypomineralization (MIH) in 8–12 year-old children living in Chandigarh, India. European Archives of Paediatric Dentistry 2022;23:97–107. https://doi.org/10.1007/S40368-021-00665-8/TABLES/3.

[133] Mahoney E, Morrison D. Further examination of the prevalence of MIH in the Wellington region. N Z Dent J 2011;107:79–84.

[134] Mahoney EK, Morrison DG. Further examination of the prevalence of MIH in the Wellington region. New Zealand Dental Journal 2011;107:79–84.

[135] Shin J-H, An U-J, Kim S, Jeong T-S. THE PREVALENCE OF MOLAR INCISOR HYPOMINERALIZATION AND STATUS OF FIRST MOLARS IN PRIMARY SCHOOL CHILDREN. Journal of the Korean Academy of Pediatric Dentistry 2010.

[136] Zawaideh FI, Al-Jundi SH, Al-Jaljoli MH. Molar incisor hypomineralisation: prevalence in Jordanian children and clinical characteristics. Eur Arch Paediatr Dent 2011;12:31–6. https://doi.org/10.1007/BF03262776.

[137] Zagdwon A, Toumba KJ, Curzon M. The prevalence of developmental enamel defects in permanent molars in a group of English school children - PubMed. Eur J Paediatr Dent 2022;3:91–6.

[138] Yi X, Chen W, Liu M, Zhang H, Hou W, Wang Y. Prevalence of MIH in children aged 12 to 15 years in Beijing, China. Clin Oral Investig 2021;25:355–61. https://doi.org/10.1007/S00784-020-03546-4.

[139] Yannam SD, Amarlal D, Rekha CV. Prevalence of molar incisor hypomineralization in school children aged 8-12 years in Chennai. J Indian Soc Pedod Prev Dent 2016;34:134–8. https://doi.org/10.4103/0970-4388.180438.

[140] Wuollet E, Laisi S, Salmela E, Ess A, Alaluusua S. Molar–incisor hypomineralization and the association with childhood illnesses and antibiotics in a group of Finnish children. Acta Odontol Scand 2016;74:416–22. https://doi.org/10.3109/00016357.2016.1172342.

[141] Wuollet E, Laisi S, Salmela E, Ess A, Alaluusua S. Background factors of molar-incisor hypomineralization in a group of Finnish children. Acta Odontol Scand 2014;72:963–9. https://doi.org/10.3109/00016357.2014.931459.

[142] Wogelius P, Viuff JH, Haubek D. Use of asthma drugs and prevalence of molar incisor hypomineralization. Int J Paediatr Dent 2020;30:734–40. https://doi.org/10.1111/IPD.12655.

[143] Wogelius P, Haubek D, Nechifor A, Nørgaard M, Tvedebrink T, Poulsen S. Association between use of asthma drugs and prevalence of demarcated opacities in permanent first molars in 6-to-8-year-old Danish children. Community Dent Oral Epidemiol 2010;38:145–51. https://doi.org/10.1111/J.1600-0528.2009.00510.X.

[144] Wogelius P, Haubek D, Poulsen S. Prevalence and distribution of demarcated opacities in permanent 1st molars and incisors in 6 to 8-year-old Danish children. Acta Odontol Scand 2008;66:58–64. https://doi.org/10.1080/00016350801926941.

[145] Weerheijm KL, Groen HJ, Beentjes VEVM, Poorterman JHG. Prevalence of cheese molars in 11-year-old Dutch children. J Dent Child 2001;68:259–62.

[146] Villanueva-Gutiérrez T, Irigoyen-Camacho ME, Castaño-Seiquier A, Zepeda-Zepeda MA, Sanchez-Pérez L, Frechero NM. Prevalence and Severity of Molar-Incisor Hypomineralization, Maternal Education, and Dental Caries: A Cross-Sectional Study of Mexican Schoolchildren with Low Socioeconomic Status. J Int Soc Prev Community Dent 2019;9:513–21. https://doi.org/10.4103/JISPCD.JISPCD_130_19.

[147] Vicioni-Marques F, Carvalho MR, Raposo F, de Paula-Silva FWG, de Queiroz AM, Leal SC, et al. Association of dental hypersensitivity and anxiety in children with molar-incisor hypomineralisation (MIH). European Archives of Paediatric Dentistry 2023;24:313–9. https://doi.org/10.1007/S40368-023-00803-4/TABLES/3.

[148] Verma S, Dhinsa K, Tripathi AM, Saha S, Yadav G, Arora D. Molar Incisor Hypomineralization: Prevalence, Associated Risk Factors, Its Relation with Dental Caries and Various Enamel Surface Defects in 8-16-year-old Schoolchildren of Lucknow District. Int J Clin Pediatr Dent 2022;15:1–8. https://doi.org/10.5005/JP-JOURNALS-10005-2088.

[149] Vanhée T, Poncelet J, Cheikh-Ali S, Bottenberg P. Prevalence, Caries, Dental Anxiety and Quality of Life in Children with MIH in Brussels, Belgium. Journal of Clinical Medicine 2022, Vol 11, Page 3065 2022;11:3065. https://doi.org/10.3390/JCM11113065.

[150] Tourino LFPG, Corrêa-Faria P, Ferreira RC, Bendo CB, Zarzar PM, Vale MP. Association between Molar Incisor Hypomineralization in Schoolchildren and Both Prenatal and Postnatal Factors: A Population-Based Study. PLoS One 2016;11:e0156332. https://doi.org/10.1371/JOURNAL.PONE.0156332.

[151] Thakur H, Kaur A, Singh N, Singh R, Kumar S. Prevalence and Clinical Characteristics of Molar-Incisor Hypomineralization in 8-16-year-old Children in Industrial Town of Solan District of Himachal Pradesh. Int J Clin Pediatr Dent 2020;13:230–4. https://doi.org/10.5005/JP-JOURNALS-10005-1767.

[152] Rehaman T, Ravichandra KS, Muppa R, Srikanth K, Kantipudi MJN, Chaitanya Ram K. Molar Incisor Hypomineralization Prevalence in the Schoolchildren of Gannavaram Mandal, Krishna District, Andhra Pradesh, India: A Cross-sectional Study. Int J Clin Pediatr Dent 2021;14:737–40. https://doi.org/10.5005/JP-JOURNALS-10005-2097.

[153] Subramaniam P, Gupta T, Sharma A. Prevalence of molar incisor hypomineralization in 7–9-year-old children of Bengaluru City, India. Contemp Clin Dent 2016;7:11. https://doi.org/10.4103/0976-237X.177091.

[154] Soviero V, Haubek D, Trindade C, Da Matta T, Poulsen S. Prevalence and distribution of demarcated opacities and their sequelae in permanent 1st molars and incisors in 7 to 13-year-old Brazilian children. Acta Odontol Scand 2009;67:170–5. https://doi.org/10.1080/00016350902758607.

[155] Souza JF, Jeremias F, Costa-Silva CM, Santos-Pinto L, Zuanon ACC, Cordeiro RCL. Aetiology of molar-incisor hypomineralisation (MIH) in Brazilian children. Eur Arch Paediatr Dent 2013;14:233–8. https://doi.org/10.1007/S40368-013-0054-3.

[156] Sosa-Soto J, Padrón-Covarrubias AI, Márquez-Preciado R, Ruiz-Rodríguez S, Pozos-Guillén A, Pedroza-Uribe IM, et al. Molar incisor hypomineralization (MIH): prevalence and degree of severity in a Mexican pediatric population living in an endemic fluorosis area. J Public Health Dent 2022;82:3–10. https://doi.org/10.1111/JPHD.12446.

[157] Sönmez H, Yıldırım G, Bezgin T. Putative factors associated with molar incisor hypomineralisation: an epidemiological study. Eur Arch Paediatr Dent 2013;14:375–80. https://doi.org/10.1007/S40368-013-0012-0.

[158] Shah VU, Dave BH, Chari DN, Shah KA. Prevalence, Severity and Associated Risk Indicators of Molar Incisor Hypomineralization amongst 8–13-year-old Children of Vadodara District Gujarat: A Cross-sectional Study. Int J Clin Pediatr Dent 2023;16:280. https://doi.org/10.5005/JP-JOURNALS-10005-2570.

[159] Schmalfuss A, Stenhagen KR, Tveit AB, Crossner CG, Espelid I. Canines are affected in 16-year-olds with molar-incisor hypomineralisation (MIH): an epidemiological study based on the Tromsø study: “Fit Futures.” Eur Arch Paediatr Dent 2016;17:107–13. https://doi.org/10.1007/S40368-015-0216-6.

[160] Salem K, Aziz D, Asadi M. Prevalence and Predictors of Molar Incisor Hypomineralization (MIH) among Rural Children in Northern Iran. Iran J Public Health 2016;45:1528.

[161] Saldarriaga A, Rojas-Gualdrón D, Restrepo M, Santos-Pinto L, Jeremias F. Dental fluorosis severity in children 8-12 years old and associated factors. Acta Odontol Latinoam 2021;34:156–65. https://doi.org/10.54589/AOL.34/2/156.

[162] Saitoh M, Nakamura Y, Hanasaki M, Saitoh I, Murai Y, Kurashige Y, et al. Prevalence of molar incisor hypomineralization and regional differences throughout Japan. Environ Health Prev Med 2018;23:1–6. https://doi.org/10.1186/S12199-018-0748-6/FIGURES/3.

[163] Rodríguez-Rodríguez M, Carrasco-Colmenares W, Ghanim A, Natera A, Acosta-Camargo MG. Prevalence and Distribution of Molar Incisor Hypomineralization in children receiving dental care in Caracas Metropolitan Area, Venezuela. Acta Odontol Latinoam 2021;34:104–12. https://doi.org/10.54589/AOL.34/2/104.

[164] Robles MJ, Ruiz M, Bravo-Perez M, González E, Peñalver MA. Prevalence of enamel defects in primary and permanent teeth in a group of schoolchildren from Granada (Spain). Med Oral Patol Oral Cir Bucal 2013;18. https://doi.org/10.4317/MEDORAL.18580.

[165] Reyes MRT, Fatturi AL, Menezes JVNB, Fraiz FC, da Silva Assunção LR, de Souza JF. Demarcated opacity in primary teeth increases the prevalence of molar incisor hypomineralization. Braz Oral Res 2019;33:e048. https://doi.org/10.1590/1807-3107BOR-2019.VOL33.0048.

[166] Reis P, Jorge R, Americano G, Pontes N, Peres A, Oliveira A, et al. Prevalence and Severity of Molar Incisor Hypomineralization in Brazilian Children. Pediatr Dent 2021;43:270–5.

[167] Ravindran R, Saji AM. Prevalence of the developmental defects of the enamel in children aged 12–15 years in Kollam district. J Int Soc Prev Community Dent 2016;6:28. https://doi.org/10.4103/2231-0762.175407.

[168] Raposo F, De Carvalho Rodrigues AC, Lia ÉN, Leal SC. Prevalence of Hypersensitivity in Teeth Affected by Molar-Incisor Hypomineralization (MIH). Caries Res 2019;53:424–30. https://doi.org/10.1159/000495848.

[169] Rai PM, Jain J, Raju AS, Nair RA, Shashidhar K, Dsouza S. Prevalence of Molar Incisor Hypomineralization among School Children Aged 9 to 12 Years in Virajpet, Karnataka, India. Open Access Maced J Med Sci 2019;7:1042–6. https://doi.org/10.3889/OAMJMS.2019.224.

[170] Rai A, Singh A, Menon I, Singh J, Rai V, Aswal GS. Molar Incisor Hypomineralization: Prevalence and Risk Factors Among 7-9 Years Old School Children in Muradnagar, Ghaziabad. Open Dent J 2018;12:714–22. https://doi.org/10.2174/1745017901814010714.

[171] Shrestha R, Upadhaya S, Bajracharya M. Prevalence of molar incisor hypomineralisation among school children in Kavre. Kathmandu University Medical Journal 2014;12:38–42. https://doi.org/10.3126/KUMJ.V12I1.13631.
